# Systematic review of the status of veterinary epidemiological research in two species regarding the FAIR guiding principles

**DOI:** 10.1186/s12917-021-02971-1

**Published:** 2021-08-11

**Authors:** Anne Meyer, Céline Faverjon, Miel Hostens, Arjan Stegeman, Angus Cameron

**Affiliations:** 1 Ausvet Europe, 3 rue Camille Jordan, 69001 Lyon, France; 2 Department of Farm Animal Health, Utrecht University, 3512 JE Utrecht, the Netherlands

**Keywords:** Veterinary epidemiology, FAIR, Data access, Salmonids, Dairy cattle

## Abstract

**Background:**

The FAIR (Findable, Accessible, Interoperable, Reusable) principles were proposed in 2016 to set a path towards reusability of research datasets. In this systematic review, we assessed the FAIRness of datasets associated with peer-reviewed articles in veterinary epidemiology research published since 2017, specifically looking at salmonids and dairy cattle. We considered the differences in practices between molecular epidemiology, the branch of epidemiology using genetic sequences of pathogens and hosts to describe disease patterns, and non-molecular epidemiology.

**Results:**

A total of 152 articles were included in the assessment. Consistent with previous assessments conducted in other disciplines, our results showed that most datasets used in non-molecular epidemiological studies were not available (i.e., neither findable nor accessible). Data availability was much higher for molecular epidemiology papers, in line with a strong repository base available to scientists in this discipline. The available data objects generally scored favourably for Findable, Accessible and Reusable indicators, but Interoperability was more problematic.

**Conclusions:**

None of the datasets assessed in this study met all the requirements set by the FAIR principles. Interoperability, in particular, requires specific skills in data management which may not yet be broadly available in the epidemiology community. In the discussion, we present recommendations on how veterinary research could move towards greater reusability according to FAIR principles. Overall, although many initiatives to improve data access have been started in the research community, their impact on the availability of datasets underlying published articles remains unclear to date.

**Supplementary Information:**

The online version contains supplementary material available at 10.1186/s12917-021-02971-1.

## Background

The FAIR (Findable, Accessible, Interoperable, Reusable) guiding principles were first published in 2016, providing a foundation to support increased re-use of scientific data [1]. Mons et al. [2] highlighted that FAIR is a continuum, which they represented with six levels, from “re-useless data” to “FAIR data with open access and functionally linked”. The ultimate goal of this set of principles is for research objects (such as datasets) to be rendered reusable and increasingly re-used. A number of articles on the topic of FAIR principles, from the original group of authors and others have been published since 2017. Jacobsen et al. [3] described some of the challenges and opportunities for implementation of each of the FAIR principles, while Thompson et al. [4] outlined the tools and technologies that are already available to support the adoption of FAIR data management, as well as the functionalities which are still lacking in that respect. Such resources enable scientific communities to identify existing solutions before considering developing their own.

There is however little visibility on the progress achieved by researchers in specific disciplines since the publication of the FAIR foundational paper. At the time of writing, very few published evaluations of FAIRness in publications and datasets were found in the literature. Van Reisen et al. [5] reviewed the implementation of the FAIR principles reported in 100 randomly selected academic journal articles citing the foundational FAIR paper. They point out that life sciences represent the vast majority of the implementation, with 95 of the selected papers related to this discipline and a very limited representation (5 papers) of the other disciplines such as social science, humanities and other sciences. As supplementary materials to their publication on FAIR metric development, Wilkinson et al. [6] also provided evaluation results for ten digital resources, as example application of these metrics. In addition, two reviews on application of FAIR principles, in Europe and Africa respectively, have been published recently, gathering large scale initiatives which are representing important steps to optimise data management and stewardship, and therefore, strive towards more FAIRness [7, 8]. These reviews highlighted initiatives in humanities, environmental science, materials science and digital health, but none in many other sectors, such as veterinary research.

The importance of veterinary research in general, and especially veterinary epidemiology, has been better acknowledged since the beginning of the COVID-19 pandemic. Managing and improving animal health is not only critical to provide a secure access to safe animal-sourced foods, but also as a key component of the global health ecosystem. The purpose of the work presented in this manuscript is to fill the current gap of knowledge about the adoption of FAIR principles in this discipline. Our study is based on a systematic review of the FAIRness of datasets associated with peer-reviewed articles relating to veterinary epidemiology research and published since 2017. The objectives are (i) to assess the state of FAIRness in this discipline, currently and over the past few years, and (ii) to explore how veterinary epidemiology research could move towards more re-usability, in line with the goal of the FAIR principles.

Many tools for evaluating the FAIRness of digital resources are available, in the form of questionnaires. Some of these tools were reviewed in peer-reviewed publications [4, 9], while others are referenced in online repositories such as the FAIRassist [10] and Research Data Alliance [11] repositories. Wilkinson et al. [6] in particular have proposed a framework and a first set of metrics developed for the evaluation of FAIRness. The authors subsequently proposed a second set of metrics, called maturity indicators, after including community feedback regarding the first set and gaining a better understanding on how data generators, managers and users were addressing FAIR principles [12]. These maturity indicators are registered by *FAIRsharing* (www.fairsharing.org), an online register of metadata standards to allow scientists to use frameworks which have been thoroughly documented. In the present manuscript, we applied the maturity indicators and tools proposed by these authors to conduct our evaluations. This framework intends to evaluate the overall maturity of an approach by assessing the FAIR maturity indicators separately and identifying specific points that can be improved, rather than evaluating the resource with a summary score of FAIRness: “FAIRness is not a competition, rather, FAIRness refers to a maturation process where digital objects are rendered increasingly self-descriptive to the machine” [12].

Given the diversity found in the animal production sector in terms of species, further definition of the scope of this study is required. Aquaculture is an increasingly important provider of animal protein worldwide [13]. There are dozens of diseases which affect the economic sustainability of aquaculture enterprises [14] and may be of concern regarding public health [15]. In this context, epidemiological studies provide key tools to better understand the complex systems in which fish and other aquatic species are produced [16], by looking at the variations in disease risk in populations and considering the interplay between host, pathogen and environment factors. Epidemiological research to improve aquatic health management, and salmonid health in particular, emerged as a discipline in the early 2000s and is still growing [17, 18]. Salmonids (including several salmon and trout species) are an important group in seawater fish production globally, with over 3 million tons produced in 2016 [13], and also an interesting group for the present work given the increasingly common use of data routinely generated by commercial producers for research. Such data are likely to be considered as confidential information, due to animal health and production data revealing production practices, adding another layer of complexity in terms of FAIRness. Given the growing importance of this sector, this study focuses on the current state of FAIRness in salmonid epidemiology.

In contrast, milk and dairy products are contributing to a much larger proportion of food produced from animals worldwide than fish [19], making dairy cattle one of the major livestock production systems. However, there are increasing concerns about the impact of terrestrial livestock production systems on the environment and on climate change. It is estimated that 15% of human-induced emissions of greenhouse gases worldwide are attributable to livestock production, with a large proportion of these emissions due to ruminants [20]. Nonetheless, ruminants, and dairy cattle in particular, remain critical in many ecosystems given their unique ability to convert feedstuffs with little nutritional value for humans into high-quality protein [21]. In this context, epidemiological research in dairy cattle is a long-established discipline, as improving animal health is key to optimise yields and thus make the most of available resources. Given the importance of dairy cattle in epidemiological research, we chose to compare our snapshot of the state of FAIRness in salmonid epidemiological research with a similar snapshot in this species to address the two study objectives stated above.

In this work, molecular epidemiology research, the branch of epidemiology using genetic sequences of pathogens or hosts to describe disease patterns, was considered separately from other branches of epidemiology, given the differences in the types of data that are collected and analysed in these disciplines. The results presented in this manuscript concern both molecular and non-molecular epidemiology in salmon, and non-molecular epidemiology only in dairy cattle.

## Results

### Data availability in salmonid research

**Fig. 1:**
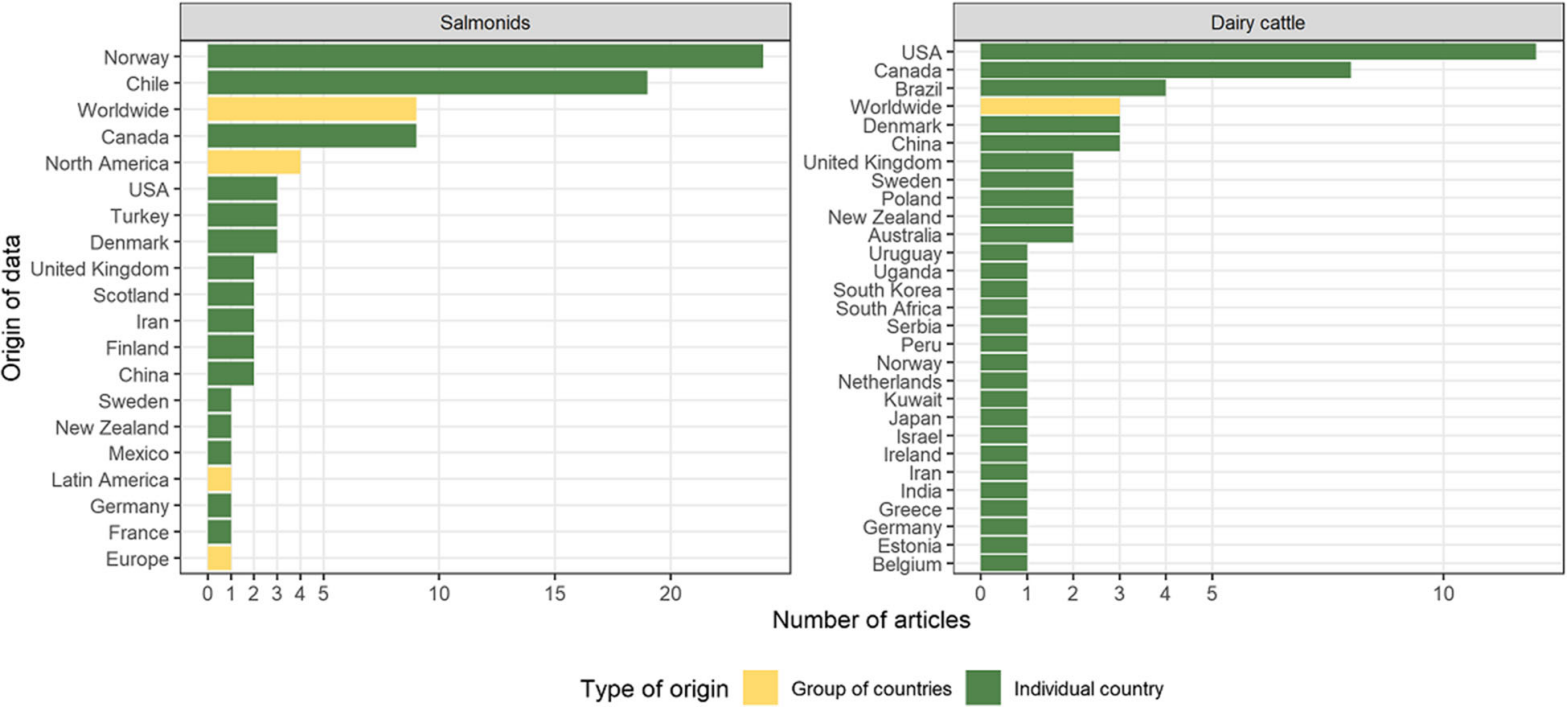
Distribution of the selected articles by country of origin and species (*N* = 152)

The literature search yielded 147 and 98 results for salmon and trout, respectively. The flow diagram of the identification, screening and inclusion of results is included in Supplementary File 1. The review process led to the inclusion of 91 articles on various epidemiological topics in salmonid production in this study (the full reference list is provided in Supplementary File 2). The selected articles presented data from 16 individual countries, with an important representation from Norway, Chile and Canada, as well as global data and grouped data from North America, Latin America and Europe (Fig. 1, left panel).

**Fig. 2:**
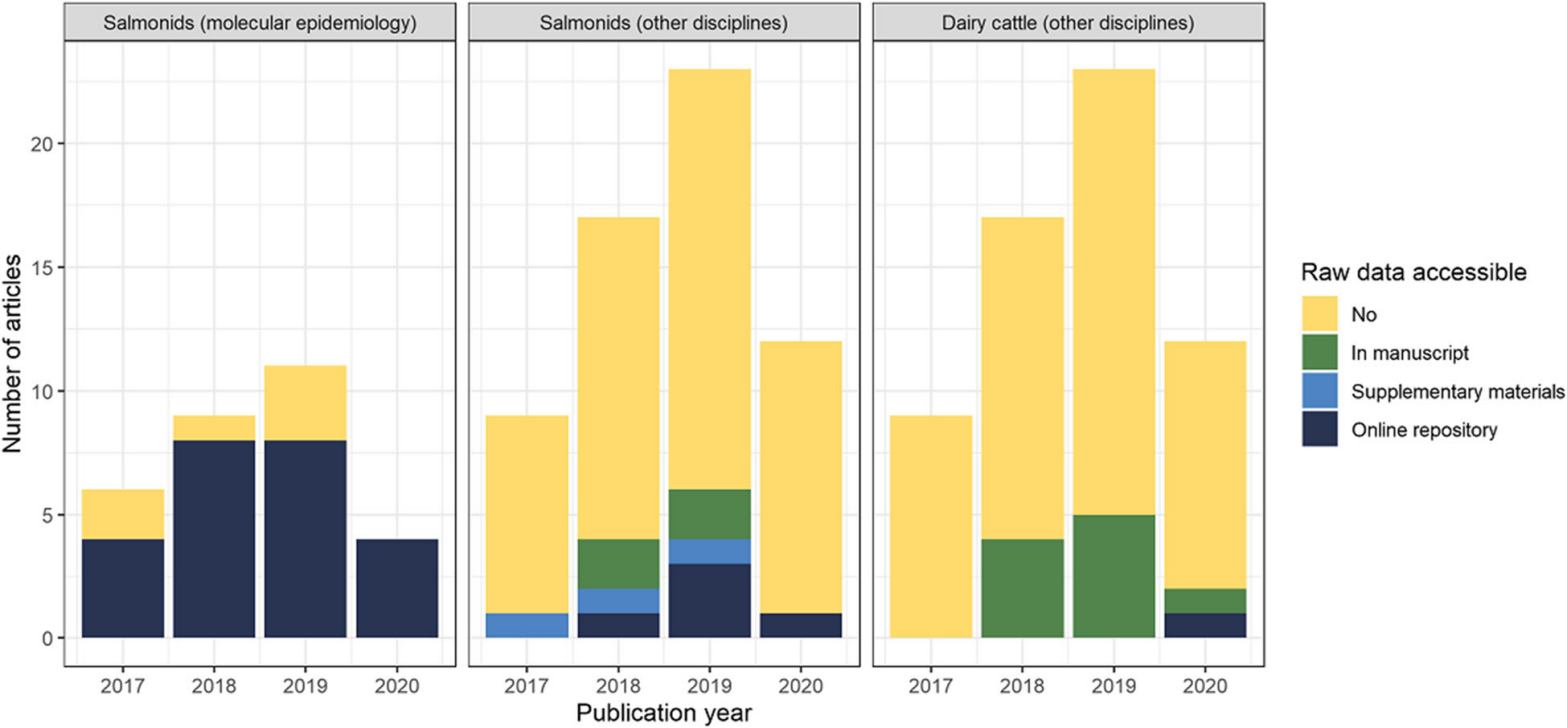
Distribution of the selected articles according to publication year, species, discipline and accessibility of raw data (*N* = 152). Note that 2020 publications were assessed until October 18th only. For the molecular epidemiology papers, the raw data referred to in this figure are the molecular data. Note that 2020 publications were assessed until October 18th only. For the molecular epidemiology papers, the raw data referred to in this figure are the molecular data

The assessment of the full texts, supplementary materials and article pages on the publisher websites showed that for 55 out of the 91 papers, the raw data supporting the work were not provided within the article, via its supplementary materials or in an online repository (Fig. 2). Raw data were available for 80% of the molecular epidemiology papers (24 out of 30) and 20% of the papers in other epidemiology sub-disciplines (12 out of 61). In addition, authors stated that raw data were available upon request in 3 out of the 55 papers for which they were not directly provided.

For the 24 molecular epidemiology papers with raw data classified as available, genetic data were uploaded in specific-purpose repositories: GenBank (19 papers), NCBI’s Sequence Read Archive (4 papers), PubMLST (3 papers) and the European Nucleotide Archive (2 papers), or in a generic-purpose repository (FigShare, 2 papers). The sum of the numbers in brackets is larger than 24 as six papers deposited data in two repositories. Genetic data were not available for the six remaining papers. Epidemiological data on the isolates or samples were also provided in two thirds of molecular epidemiology papers (21 out of 30). Such data were generally shared as tables within the manuscript itself (14 papers), and/or as supplementary materials (either as PDF tables, 3 papers, or Word tables, 5 papers). Isolate data were shared in a MicroReact project for one paper and could be downloaded as a tab-separated file.

For the 12 papers in other epidemiology sub-disciplines for which raw data were available, these were deposited in an online repository in 5 cases: a generic-purpose repository (Mendeley Data, Dryad) or institutional repositories (Marine Data BC and Norwegian Marine Data Centre). In the other cases, the raw data were shared in the manuscript itself as Tables (4 papers), as supplementary materials (either as PDF tables, 1 paper, or Excel file, 2 papers). For two of these papers, it appeared that only part of the data used to conduct the work was made available. Last, ten of the 61 non-molecular epidemiology papers used commercial data, but raw data were available for only one of these ten papers.

### Data availability in dairy cattle research

At the end of the eligibility and inclusion steps (see flow chart in Supplementary File 1), a total of 61 papers concerning epidemiology research in dairy production were randomly selected and assessed for data availability. A third of the papers related to Canada or USA (20 papers out of 61), while the remainder originated from 26 other countries or were based on worldwide data (3 papers) (Fig. 1, right panel). The assessment of dairy papers showed that raw data were available for 18% of them (11 out of 61) (Fig. 2). In ten of those, the raw data were provided within the manuscript. Raw data were deposited in a generic-purpose repository for the remaining paper (Scholars Portal Dataverse). In addition, authors stated that raw data were available upon request in 3 out of the 50 papers for which they were not directly provided. Four of the 61 dairy cattle papers used commercial data, but raw data were not available for any of these four papers. An overview of the data availability in both species is presented in Table 1.

**Table 1:** Overview of data availability in articles included in this review

Species (discipline)	Salmonids (molecular epidemiology)	Salmonids (other disciplines)	Dairy cattle (other disciplines)
**Number of articles included in the review**	30	61	61
**Number of articles which had raw data available**	24	12	11
**• In manuscript**	0	4	10
**• As supplementary materials**	0	3	0
**• In an online repository**	24	5	1
**Number of datasets included in the FAIRness assessment**	30	8	1

### Data availability statements

**Fig. 3:**
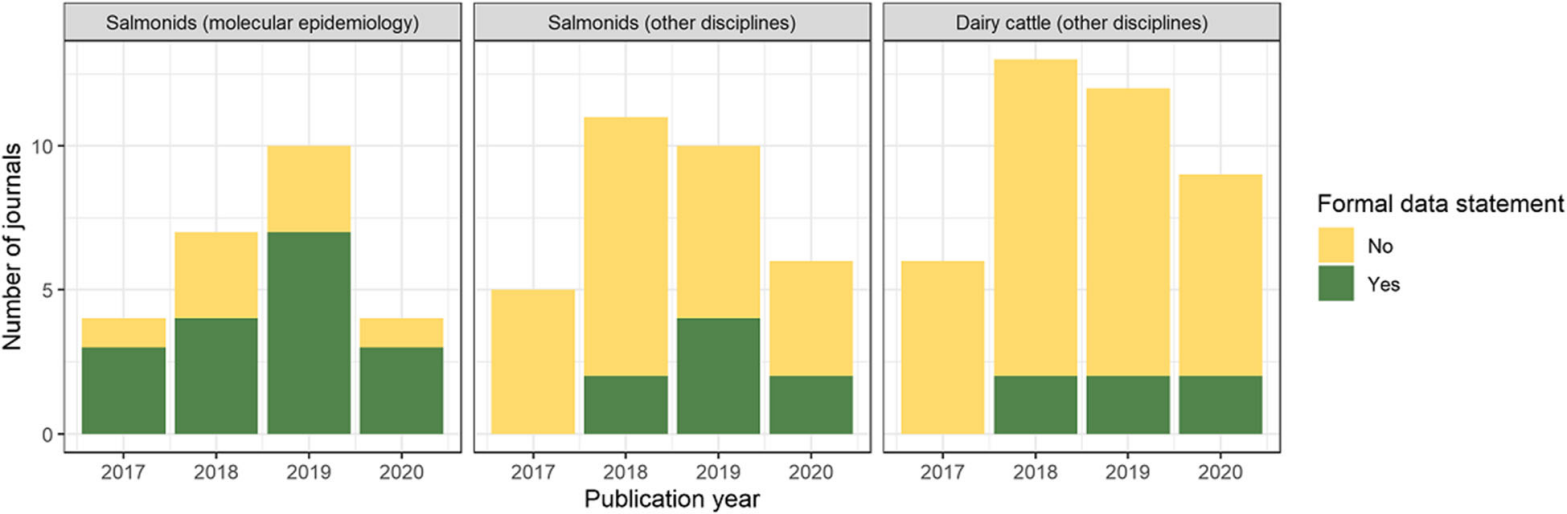
Presence of a data availability statement in the journals (*N* = 62) publishing the selected articles according to publication year and species. Some individual journals may appear in more than 1 year, species or discipline. Note that 2020 publications were assessed until October 18th only

The 152 articles assessed in this part of the study were published in 60 different journals. Among those, 17 included a formal data availability statement either as a dedicated section in the manuscript or as a supplementary item (Fig. 3). Such statements were introduced during the study period in some of the journals most represented by the selected articles, such as Aquaculture, Journal of Fish Diseases and Preventive Veterinary Medicine. These additional sections may be named “Availability of data and materials”, “Data access”, “Data accessibility”, “Data availability”, “Data profile”, “Data summary” or “Research data for this article”. Among papers not related to molecular epidemiology, data availability statements were provided in 6 and 10 of the dairy and salmonid articles, respectively. In salmonid papers, the statements mentioned that the study datasets were available upon request (2 papers), that authors did not have permission to share them (1 paper), that the data had been deposited in an online repository (4 papers), or that “all relevant data are within the paper and its Supporting Information files” (or similar, 3 papers). In two of the three latter papers, the raw data did not appear to be available despite the statement. In dairy papers, the statements mentioned that study datasets were available upon request (3 papers), that authors did not have permission to share them (2 papers), or that the data had been deposited in an online repository (1 paper).

Some individual journals may appear in more than 1 year, species or discipline. Note that 2020 publications were assessed until October 18th only.

### FAIRness assessment

Evaluation results for 13 maturity indicators of the available datasets are presented in Table 2 (papers on molecular epidemiology) and Table 3 (other papers). The assessment criteria are described in the Methods section (Table 4). A number of papers provided the data used in the study within the manuscript itself (text or tables) (Fig. 2), including 4 and 10 papers on salmonids and dairy cattle, respectively. Those datasets were not evaluated for FAIRness as they do not form a distinct digital resource from the article itself.

**Table 2:** Evaluation of FAIRness for 30 datasets accompanying 24 articles in molecular epidemiology of salmonids

Maturity indicator assessment													Source article
F1.1	F1.2	F2	F3	F4	A1	A2	I1	I2	I3	R1.1	R1.2	R1.3	
0	0	2	0	2	2	0	1	2	2	2	2	0	[22] (PubMLST) + [23] (PubMLST) + [24] (PubMLST)
2	0	2	2	2	2	2	1	2	2	1	2	2	[22] (European Nucleotide Archive) + [25] (European Nucleotide Archive)
2	0	2	2	2	2	0	1	2	0	1	0	2	[26, 27] + [28] (Sequence Read Archive)
2	0	2	2	2	2	0	1	2	0	1	2	2	[29–35] + [23] (GenBank) + [24] (GenBank) + [28] (GenBank)
2	0	2	2	2	2	0	1	2	2	1	2	2	[36–43] + [44] (GenBank)
2	2	1	2	2	2	2	1	0	0	2	0	2	[45]
2	2	1	2	2	2	2	1	2	0	2	0	2	[25] (FigShare)
NE	NE	NE	NE	NE	NE	NE	NE	NE	NE	NE	NE	NE	[44] (Sequence Read Archive)

The scores correspond to whether a given maturity indicator was met (2), partially met (1) and not met (0), respectively (cf. Table 4). The mention NE corresponds to a resource which could not be evaluated given that the dataset accession number was invalid. Datasets which have obtained the same combination of scores on the 13 indicators are grouped in the same row. Given that six source articles had two datasets associated with them (deposited in separate repositories), their reference appears twice in this table. To distinguish them, the corresponding repository is indicated in brackets after the reference.

**Table 3:** Evaluation of FAIRness for nine datasets accompanying articles in non-molecular epidemiology papers

Maturity indicator assessment													Source article
F1.1	F1.2	F2	F3	F4	A1	A2	I1	I2	I3	R1.1	R1.2	R1.3	
2	2	0	0	2	2	0	0	0	0	2	0	0	[46]
2	2	2	2	2	2	2	1	1	0	2	2	2	[47]*
0	0	0	0	0	2	0	0	0	0	2	2	0	[48]
2	2	2	2	2	2	2	0	0	0	2	2	2	[49]
2	2	2	2	2	2	0	0	1	0	2	2	2	[50]
2	0	2	2	2	2	0	0	1	0	2	2	0	[51]
2	2	2	2	2	2	2	0	0	0	2	2	2	[52]
2	2	2	2	2	2	0	1	0	0	2	2	2	[53]
0	0	0	0	0	2	0	0	0	0	2	0	0	[54]

The scores correspond to whether a given maturity indicator was met (2), partially met (1) and not met (0), respectively (cf. Table 4). The mention NE corresponds to a resource which could not be evaluated given that the dataset accession number was invalid. Eight papers concerned salmonids and one dairy cattle (the latter is marked with an asterisk).

**Table 4:** List of 13 FAIRness maturity indicators evaluated in this study, based on the framework proposed by Wilkinson et al. [6, 12]

			Maturity levels
Indicator identifier	Indicator name	Indicator description	
F1.1	Identifier uniqueness	Whether there is a scheme to uniquely identify the digital resource	0: Indicator is not met 2: Indicator is met
F1.2	Identifier persistence	Whether there is a policy or scheme which ensures the persistence of the digital resource identifier	0: Indicator is not met 2: Indicator is met
F2	Data are described with metadata	Whether metadata corresponding to the digital resource are available	0: Indicator is not met 2: Indicator is met
F3	Resource identifier in metadata	Whether the metadata contains the unique identifier for the digital resource	0: Indicator is not met 2: Indicator is met
F4	Indexed in a searchable resource	Whether the digital resource can be found by web-based search engines using search terms such as title, author or key words. Google Search was used in this assessment	0: Indicator is not met 2: Indicator is met
A1	Access protocol	Whether there is an open and free access protocol to retrieve the digital resource, and if not, whether the specifications to access restricted content are provided	0: Indicator is not met 2: Indicator is met
A2	Metadata longevity	Whether there is a policy to guarantee the persistence of metadata even in the case of absence or removal of the digital resource itself	0: Indicator is not met 2: Indicator is met
I1	Use a knowledge representation language	Whether a formal language for knowledge representation is used in the digital resource. This indicator was assessed in terms of the format of the data.	0: Indicator is not met 1: Indicator is partially met when the resource is in a structured, non-proprietary, editable format (e.g., CSV, XML) 2: Indicator is met when the language used is cited and documented
I2	Use of FAIR vocabularies	Whether the digital resource uses formal and shared vocabularies (ontologies) for knowledge representation, which are themselves terms from open, community-accepted vocabularies published in an appropriate knowledge-exchange format.	0: Indicator is not met 1: Indicator is partially met when the vocabularies used in the resource are documented 2: Indicator is met
I3	Use of qualified references	Whether the digital resource or its metadata contain relationships with third-party data, with an explicit and useful semantic meaning	0: Indicator is not met 2: Indicator is met
R1.1	Accessible usage license	Whether there is a license document for the digital resource and the ability to retrieve those documents	0: Indicator is not met 1: Indicator is partially met when elements concerning the conditions for re-use, copying or distributing the resource are available but no formal license can be found 2: Indicator is met
R1.2	Detailed provenance	Whether the digital resource content is associated with provenance information associated with the data, covering at least: (i) who produced the data and when, and (ii) why and how the data was produced (context and relevance of the data). The availability of such information in the digital resource itself or its metadata was evaluated, not in the content of the associated article	0: Indicator is not met 2: Indicator is met
R1.3	Meet community standards	Whether the digital resource is listed by a recognized body as meeting community standards. Repositories were considered as compliant when listed by FAIRsharing, the Registry of Research Data Repositories or Core Trust Seal (www.coretrustseal.org)	0: Indicator is not met 2: Indicator is met

The indicator identifiers are the same as the corresponding guiding principle identifiers for simplicity

Molecular digital resources were almost always identified by a globally unique identifier defined by the repository, but this identifier was generally not persistent (F1). Most resources were associated with metadata including the data identifier (F2 and F3) and provenance information (R1.2). All resources were indexed by a search engine (F4) and accessible via an open, free protocol (A1) but the persistence of the metadata should the resource become unavailable was not guaranteed (A2). No resources used a formal knowledge representation language, but all were provided in standardized formats, such as GenBank data (I1). Most used FAIR vocabularies (I2) and about half were linked to other relevant resources (I3). Clear license conditions were not often provided (R1.1), given that several common repositories such as GenBank state that the repository managers “cannot provide comment or unrestricted permission concerning the use of the information contained in the molecular databases” or similar. All repositories but one were certified or listed as trusted in known community schemes (R1.3). The digital resources evaluated here originated from a range of 12 individual countries and from two multi-country studies.

Within non-molecular datasets, most digital resources were identified by a globally unique and persistent identifier, generally a Digital Object Identifier (F1). Six out of nine resources were associated with metadata including the data identifier (F2 and F3) while most had provenance information (R1.2) and were indexed in a search engine (F4). Accessibility indicators were similar to those observed above for molecular data (A1, A2). No resources used a formal knowledge representation language and only two were provided in editable, non-proprietary format (I1). No resources used standardized vocabularies, and only two provided some documentation regarding the vocabularies used (I2). None were linked to other relevant resources (I3). Clear license conditions were always provided (R1.1) and five datasets were deposited in repositories certified or listed as trusted in known community schemes (R1.3). The nine digital resources which were evaluated originated from a range of countries: Canada (two datasets), Finland (one dataset), Mexico (one dataset), Norway (two datasets) and USA (one dataset) or from multi-country studies (two datasets).

Finally, the data sources for the nine non-molecular epidemiological digital resources evaluated in this section were databases from government agencies (e.g., the Directorate of Fisheries in Norway [50] and the Aquatic Health Committee of Oaxaca in Mexico [49]) or international agencies (e.g., European Community Reference Laboratory for Fish Diseases [48]), data from the published literature or data specifically generated for the purpose of the study. The dataset made available by Soler-Jiménez et al. [54] in their literature review was the only dataset from a commercial source. However, this dataset was not collected specifically for the purpose of the study described in this publication. The authors were able to obtain mortality, environmental and management data from a group of fish producers in Mexico in the context of another study, which could not be identified in peer-reviewed sources at the time of writing. The dataset contained a few hundreds of farm-level records of several variables, without associated farm identifiers.

## Discussion

Most datasets used in non-molecular epidemiological studies were not findable, or “re-useless” as characterised by Mons et al. [2]. These authors estimated that 80% of datasets in science belonged to this category. Our assessment suggests that the proportion in veterinary epidemiology is at least as high for the two species included in this work. In addition, no clear trend of improvement was observed over the past 4 years. Data availability was much higher for molecular epidemiology papers, with 80% of the articles assessed depositing genetic data in online repositories. The low proportion of raw data provided as distinct digital resources in non-molecular epidemiology publications means that only few datasets could be assessed in terms of FAIR maturity indicators in this study (nine out of 122 articles assessed). Of note, our assessment considered whether all the raw data used to produce the results were available for a given paper, but we did not try to reproduce any of the results as this was out of scope of the present study. Such additional assessment may allow the identification of papers for which some of the data required to reproduce the results were not available.

For articles with no raw data or, more rarely, data shared within the manuscript itself, the absence of separate data objects means that the FAIR assessment could not be conducted. Although these data may be reusable as they are both findable and accessible by researchers, and associated with provenance information and other metadata, their format (not directly searchable and editable) does not make them interoperable. In addition, they are neither machine-findable nor machine-accessible. Such considerations also apply to the data shared in supplementary files assessed in this study. For supplementary materials, metadata may not be readily available (F2), although researchers may manually find relevant information in the article, and these resources sometimes do not have a unique resource identifier (F1), although some journals associated a dedicated DOI to each supplementary item. Supplementary materials were not searchable in search engines, as the article itself is the object that is indexed (F4). Although providing raw data as supplementary materials does not make datasets FAIR, it is a common and practical way for researchers to respond to the increasingly pressing requests to make data available. It could be argued that there is little difference between sharing raw data in a manuscript table or in a supplementary material table and that treating them differently is arbitrary. However, a criterion for what constitutes raw data had to be established for the specific purpose of this study. In accordance with the FAIR framework which focuses on digital objects, we chose to only assess the FAIRness of raw data which were provided as separate digital resources, as described in the Methods section.

While the number of papers for which the raw data were available was similar between salmonid and dairy cattle in non-molecular epidemiology (12 and 11, out of 61, respectively), datasets were made available as individual digital resources more often by salmonid researchers than by dairy researchers (8 and 1 datasets, respectively). It was not possible to compare the FAIR maturity indicators between the two species given the small sample size and they are discussed here together. Across disciplines and without considering the case of supplementary material datasets which was already discussed above, the FAIRness assessment showed that most resources were uniquely identified, although persistence of these identifiers was generally not provided for molecular datasets (F1). Indicators F2, F3 and R1.2 were often met, with some metadata provided for the digital resource, along with the data identifier and provenance information. The persistence of the metadata should the data objects be removed was generally not guaranteed regardless of the repository considered. Importantly, all data objects were discoverable by web-based search engines (F4) and freely accessible via an open protocol (A1).

While the available objects generally scored favourably for Findable, Accessible and Reusable indicators, Interoperability was more problematic. The datasets identified in our study were not using a “formal, accessible, shared, and broadly applicable language for knowledge representation” as per the definition of the I1 indicator [1]. In non-molecular epidemiology, datasets were often shared as Excel files, with little to no documentation of the content of the different data fields, and no linked objects could be identified. Much progress remains possible on the I1, I2 and I3 indicators for these resources. Examples of existing resources which may be used to improve Interoperability in epidemiological research are the *AGROVOC* vocabulary developed and managed by the Food and Agriculture Organization (http://www.fao.org/agrovoc/) and the *SNOVET* systematized nomenclature for veterinary medicine [55]. By contrast, standardized formats (e.g., XML) and vocabularies are used in the National Centre for Biotechnology Information (NCBI)‘s databases considered in this study for molecular datasets (GenBank and Sequence Read Archive) (23 out of 30 datasets). As the I1 maturity indicator is not associated with an agreed list of what constitutes a valid language for knowledge representation for a given discipline, there is room for interpretation by the person performing the assessment in terms of defining their scoring system. As such, the scoring system used in the present study may not be valid for another study or discipline. It is important to recognize that, as the coding of molecular data is universal, it is much easier to achieve interoperability for such datasets, as shown by the good scores achieved on this indicator compared to non-molecular epidemiology. In addition, the digital archiving of such data is concentrated by a few stakeholders as NCBI is collaborating with other large repositories such as DNA DataBank of Japan and the European Nucleotide Archive, making it practical for the interested researcher to find related data, even if they are not explicitly linked (I3). Molecular epidemiology papers were also frequently providing epidemiological data (isolate or sample data) in a tabular format (70% of papers). Such data, which can be considered as raw data or as metadata for the genetic data, are critical to support reusability. Although inferior to the availability of genetic data (80%), the availability of epidemiological data was substantially higher than that observed in non-molecular papers. Finally, frequent re-use of published genetic sequences by other researchers is observed in the literature, showing the progress made in this discipline compared with other disciplines. In summary, the relatively consistent structure of molecular datasets, the availability of appropriate repositories, and the existing demand for data re-use are some of the factors which may explain the differences in data availability observed between molecular and non-molecular papers in our study.

Meeting indicators related to the Findable, Accessible and Reusable principles is likely possible for researchers who do not have specific skills in data management. On the other hand, interoperability appears to be a more complex objective. The process of migrating the Pathogen-Host Interaction Database, in plant sciences, to a FAIR-compliant form [56] illustrates that the data transformation required to apply machine-readable standards for knowledge representation require specialist knowledge in this area. This may be an obstacle for both funders and scientists who may not readily understand the concepts or have the skills required for effective data preparation, management and long-term preservation [57–59]. In this regard, more systematic data management training is needed in graduate programmes, both to develop awareness around open science and FAIRness and to teach specific skills required to reach these goals. Meanwhile, researchers may already take simple measures to increase the interoperability of their datasets. For example, documenting the content of tabular data in a systematic manner, indicating the content, type and unit of each data field, is an accessible step for rendering datasets self-descriptive, short of using knowledge representation languages and FAIR vocabularies. Units and conditions of measurement are critical for the re-use of quantitative data. In the case of ‘oxygen’ levels in fish cages, one needs to know the type and unit of the measurement (e.g., oxygen saturation in %, oxygen concentration in mg/L) as well as the depth and temperature of the measurement to allow for correct interpretation.

No comparable assessments of FAIRness in publications were found in the literature but the subjects of data sharing and open data in research have been abundantly documented. A strong contrast is observed between willingness to share research data expressed by scientists and availability of datasets in practice. Survey respondents often declare willingness to share at least some data publicly (around 80% of them depending on the discipline and study) [60, 61]. Recent studies looking at the proportion of published work for which datasets were publicly available showed relatively low levels of availability: 8% in geoscience flux research [62], less than 10% in psychology research [57], 14% in morphology research [63] and 18% in biomedical research [64]. Even in biomedical journals with a full data sharing policy for randomized controlled trials, a review found that only 17 out of 37 eligible articles satisfied the definition for data availability [65]. Our present findings are consistent with these observations. Despite the plethora of published literature on the topic of data sharing and the growing availability of technological solutions, the evolution of practices and attitudes remains slow. In this regard, as our study was conducted only 4 years after the principles were formally published, it is not surprising that no significant changes were observed yet. These results will provide a baseline measure for future evaluations aiming at identifying longer-term changes.

Regardless, some scientific disciplines are significantly ahead in terms of data sharing, for example in genomics [66], as confirmed in the present study for molecular epidemiology in animal species. In other disciplines, barriers to making research datasets available have been explored [57, 61, 67, 68] and some of the factors which can positively impact data sharing have been identified [69–71]. Top-down pressure, in the form of strong encouragement or policies of mandatory sharing, from funding bodies and journal publishers appears to be highly effective. For measurable progress, such policies and requirements must not remain theoretical but need to be verified in practice. Data management plans indicating how FAIR principles will be applied are an increasingly common requirement from research funding organisations [72, 73].

In the animal production sector in particular, data provided for research by stakeholders may be considered as confidential information, due to animal health and production data revealing production practices as well as representing commercial assets and competitive advantages. Fourteen of the 122 non-molecular epidemiology papers reviewed in this study were based on commercial data, including four in dairy cattle and ten in salmonids. The availability of raw data was very low for these potentially sensitive commercial production data (only one of the fourteen papers made the raw data publicly available). It is reasonable to assume that raw data provided by commercial producers are even more concerned by privacy issues than datasets collected by other means. In such cases, researchers are subjected to conflicting needs, with transparency, reproducibility and reusability on one side, and data confidentiality on the other side. Our results suggest that researchers may have more room to make datasets available when they collected these data from third-party sources, such as government databases holding data submitted by industry for regulatory purposes (for example the study by Myksvoll et al. [50]). Sourcing research data from third-party data integration initiatives may allow the generation of epidemiological datasets which are easier to share publicly, due to pre-existing data sharing agreements with the industry. An example of such initiative found during this review is *Fish-iTrends*, a sea lice data management system administered by the Atlantic Veterinary College in Canada [74]. Another example in salmonids is the attempt to set up a data integration platform described by Meyer et al. [75]. The use of animal production commercial data for research can significantly increase access to quality data with excellent coverage in time and space of the populations of interest, as shown by studies included in this work such as analyses conducted in the Chile salmonid industry [76, 77] and in the Canadian dairy industry [78]. The confidentiality level required for these datasets as well as barriers to data sharing related to competitiveness and anti-trust regulation aspects suggest that there could be a fundamental incompatibility between the principles of open-access data and the use of commercial data for research. However, progress towards more FAIRness does not require the datasets to be made open access. The four first levels presented by Mons et al. [2] are compatible with access-restricted datasets. Data integration systems such as those proposed above may allow progress to be made towards improved FAIRness. For example, authors making datasets findable and their metadata findable, accessible, interoperable and reusable would reach the fourth of these levels, “FAIR data with restricted access”. This would better enable other researchers to assess which digital resources could potentially be useful to them and express their interest in reusing such datasets. Negotiations regarding the conditions for access, such as the appropriate type of license, could then be undertaken between interested parties. This would represent significant progress from the “re-useless data” stage where most of the datasets assessed in the present study were found.

Finally, our assessment showed that molecular datasets appear relatively compliant with machine readability, while this was generally not the case for the nine non-molecular available datasets. Thus, machine readability remains uncommon for non-molecular epidemiological data, especially as these nine datasets only made up for a small proportion of the 61 studies initially identified. The approach proposed by Wilkinson et al. [12] puts emphasis on machine readability: “detecting and validating behaviours of digital objects that make them machine-readable and reusable”. Given the relative novelty of FAIRness implementation in veterinary epidemiology, it is not surprising that only few resources were standardized for machine readability, while most provide human-readable content. In addition, the FAIRness assessments strictly focus on the digital object itself, and therefore do not assess other aspects of compliance with generic data management good practices, such as data curation and governance. The salmonid data platforms used in some of the studies [74, 76, 77] show the progress made by producers and data users to explore new ways of managing data, which require data curation and governance aspects to be defined in a collaborative manner. The FAIR principles also do not consider discipline-specific attributes which may be considered critical. For instance, some authors proposed an extension of the FAIR principles to better address the reproducibility and privacy protection challenges encountered in health research [79]. There are no standard indicators available to assess the additional aspects, and they were not considered within the scope of this study. Last, FAIRness assessments may also be applied to model code objects, when these are shared by their authors. Although we did not specifically address this aspect in the present work, we noted that none of the fourteen mathematical modelling studies provided access to such model objects.

## Conclusion

In conclusion, we recommend that the FAIR framework is progressively integrated in the routine workflow of researchers in veterinary epidemiology, starting with more education, training and communication. Furthermore, the barriers to reach the goals of data re-usability which have been set for a few years should be identified by discipline. This would allow the design and implementation of interventions to overcome these barriers. Last, we suggest a stepwise approach to improving the FAIRness of research data, in which the first step would be to make a large proportion of datasets and their metadata findable as digital resources.

## Methods

The methods used for this systematic review are reported in accordance with the PRISMA statement [80]. A literature search was conducted on October 18th, 2020 to identify all peer-reviewed publications related to epidemiological research in salmonid production and in dairy cattle published between January 1st, 2017 to October 18th, 2020. We searched scientific literature referenced in three electronic databases: Scopus, Web of Science and PubMed. The documents retrieved were imported into a desktop reference management system for screening, eligibility assessment and further analysis.

For the salmonid dataset, we used the following keywords: (epidemiology) AND (salmon OR trout) AND (aquaculture). All titles, abstracts and key words were screened to select the results relevant to our study, i.e., articles related to epidemiological research in salmonid production. All farmed salmonid species were included (e.g., Atlantic salmon, coho salmon, rainbow trout). We excluded (i) studies in other disciplines (clinical reports, molecular biology, proteomics, bacteriology, microbiology, parasitology,^1^ phylogenetics, physiology, food safety, economics, welfare), (ii) studies not conducted in farmed salmonids (wild salmonids, other species) and (iii) documents other than peer-reviewed publications (e.g., conference papers). Three additional articles were removed at the full-text review stage (see below) as they were discussion papers and therefore not based on any formal dataset.

For the dairy cattle datasets, we used the following keywords: (epidemiology) AND (dairy) AND (cattle OR cow). Given the large number of database results, only titles and key words were screened to select the results relevant to our study, i.e., articles related to epidemiological research in dairy cattle. Abstracts were reviewed only when the title and key words did not provide sufficient information. Exclusion criteria (i) and (iii) mentioned above were used. For criterion (ii), studies not conducted in dairy cattle (e.g., dairy goats) were excluded. Additional exclusion criteria were (iv) articles which were not available in English and (v) molecular epidemiology papers. The latter were not considered for inclusion, as the comparison between salmonids and dairy cattle focused on studies in non-molecular epidemiology. Given the large number of eligible results for dairy (739 articles), we used random sampling to select full-text articles which were reviewed for data availability. The sampling was stratified by publication year (2017 to 2020), with the number of dairy papers selected each year matching the number of available salmonid papers for that year. A pseudo-random number generation function in Microsoft Excel was used for this purpose.

The full text of all articles selected for inclusion was then reviewed and assessed, along with any supplementary materials and information available on the publisher’s Web page for the article. Relevant information for our study (DOI, publication year, species, country, title, type of work and information regarding nature and availability of datasets) was extracted and tabulated. The nature of the data used in the study was classified as commercial (i.e., data collected and managed by commercial producers for their own purposes) or non-commercial data (i.e., data provided by public agencies and industry bodies, data collected from the literature and data collected on purpose for the study such as experimental data or farm survey data). Once completed, the tabulated dataset was verified against each article to identify and correct any extraction error. For salmonids, given the very different nature of datasets between disciplines, molecular epidemiology papers were assessed separately from non-molecular epidemiology papers.

Raw data were considered ‘available’ when they were provided in the manuscript’s main text, as supplementary materials or in an online repository. Data available upon request to the authors were not considered as available in this study. Data considered as raw data in this assessment were non-aggregated data, provided at the level to which they were collected (i.e., excluding any summary data). For mathematical modelling studies (simulation studies), the model inputs and parameter values were considered as the raw data for the purpose of this assessment, not the model outputs such as simulation results for example. The rationale for identifying ‘available’ datasets in this first stage was that these data are potentially findable and accessible outside of the research team which produced them. In some cases, all the data required to reproduce the results may have been provided in the manuscript itself (either in the text or as tables in the article). Such data could not be evaluated for FAIRness as they did not form a distinct digital resource from the article itself. Therefore, they were excluded from the next stage of the assessment described in the next paragraph.

The information required to assess FAIRness was then extracted for each digital resource identified as available. Furthermore, information about the online repositories used to deposit datasets by the articles’ authors was obtained from the Registry of Research Data Repositories (www.re3data.org). A list of 13 FAIR maturity indicators relevant for our work was compiled based on the indicators proposed by Wilkinson et al. [6, 12] (Table 4). Maturity indicators should be applied to a single digital resource, and therefore the target resource must be defined clearly, as some of the principles apply to both the data and the associated metadata [1]. Here, the resources we evaluated were the datasets associated with each paper and identified during the first steps of the work. For molecular epidemiology papers, the datasets of interest were those containing genetic data rather than the sample or isolate data. While the authors of this framework have used a binary scoring system (pass or fail) in their maturity indicator tests (w3id.org/AmIFAIR), we felt that some resources presented some maturity regarding a certain indicator while not entirely meeting the requirement. Therefore, resources were assessed with a 3-level scoring system (2, 1 or 0), according to whether they fully met, partially met or did not meet the requirements for each indicator, respectively. After completion of the assessment, the scores were reviewed individually against each digital resource to identify and correct any error.

The assessment of articles and datasets described above was conducted by the first author (AM), while a parallel assessment of 10% of the items included in the study was conducted by one of the co-authors (CF). At the article level, the availability of raw data, data statement and type of data were assessed by CF for 15 randomly selected articles. A minor discordance was noted for 3 of these articles and assigned to the way these items were assessed. The present Methods section was clarified accordingly. At the dataset level, the FAIRness scores were assessed by CF for 8 randomly selected datasets. Different scores were attributed to 8 indicators out of the 104 indicators which were doubly assessed (13 indicators by dataset). Four of these differences were for the indicator I1, and one each for indicators F1.1, F3, A2 and R1. Based on these differences, the maturity levels for these indicators were re-evaluated and re-defined (Table 4) by the two researchers, and all datasets were re-assessed based on these changes.

## Supplementary Information

**Additional file 1.** Literature search flow diagrams.

**Additional file 2.** Full reference list of the literature review.

## Data Availability

The dataset supporting the conclusions of this article is available in the FigShare repository under the following collection: 10.6084/m9.figshare.c.5316863.

## Footnotes

1 papers looking at epidemiology-related aspects of sea lice were retained due to high relevance of this species for the aquaculture sector.
