# Supplementary material for: Systematic review of the status of veterinary epidemiological research in two species regarding the FAIR guiding principles
Source: BMC Vet Res. 2021 Aug 11;17:270. doi: 10.1186/s12917-021-02971-1 (PMC8355576; doi:10.1186/s12917-021-02971-1)
Supplement: Supplementary file 1 — Additional file 1. Literature search flow diagrams. [file 12917_2021_2971_MOESM1_ESM.pdf]

Additional file 1 Literature search flow diagrams

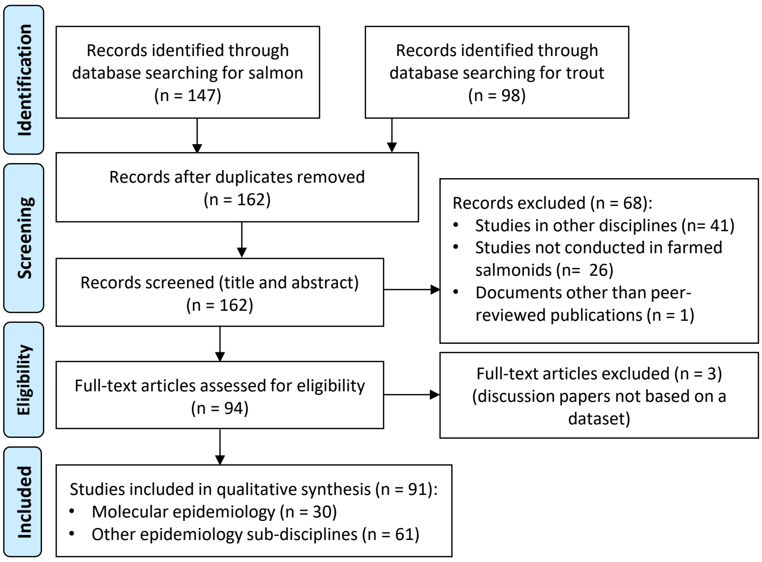

Figure 1. PRISMA flow diagram of the literature search for salmonids.

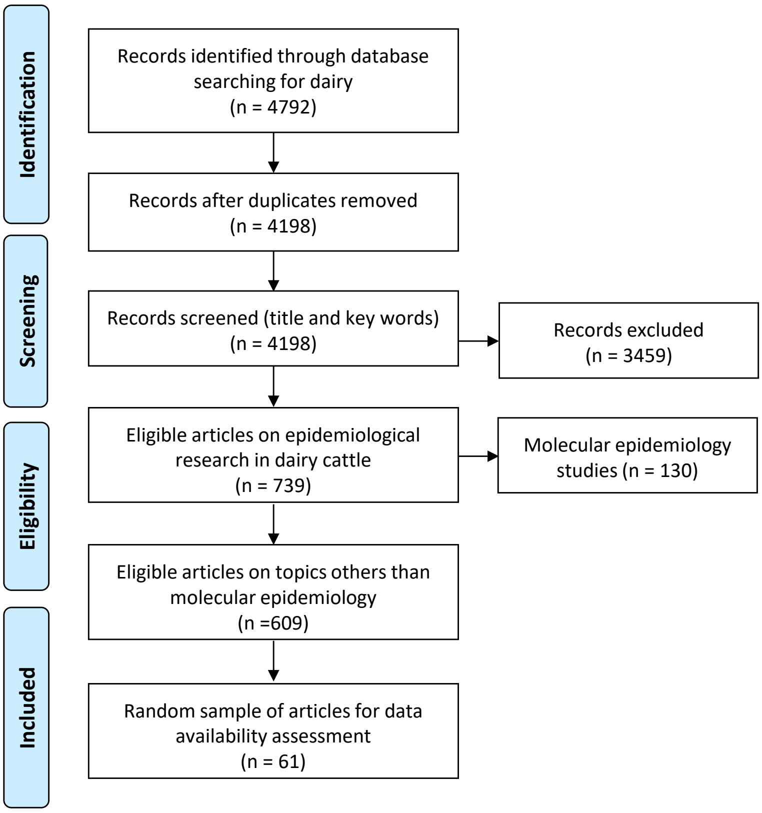

Figure 2. PRISMA flow diagram of the literature search for dairy cattle.
