# Supplementary material for: Systematic review of the status of veterinary epidemiological research in two species regarding the FAIR guiding principles
Source: BMC Vet Res. 2021 Aug 11;17:270. doi: 10.1186/s12917-021-02971-1 (PMC8355576; doi:10.1186/s12917-021-02971-1)
Supplement: Supplementary file 2 — Additional file 2. Full reference list of the literature review. [file 12917_2021_2971_MOESM2_ESM.pdf]

Additional file 2 Full reference list of the literature review

Adamek, M., Hellmann, J., Flamm, A., Teitge, F., Vendramin, N., Fey, D., Riße, K., Blakey, F., Rimstad, E., Steinhagen, D., 2019. Detection of piscine orthoreoviruses (PRV-1 and PRV-3) in Atlantic salmon and rainbow trout farmed in Germany. Transbound. Emerg. Dis. 66, 14–21. https://doi.org/10.1111/tbed.13018

Adams, A.E., Lombard, J.E., Fossler, C.P., Román-Muñiz, I.N., Kopral, C.A., 2017. Associations between housing and management practices and the prevalence of lameness, hock lesions, and thin cows on US dairy operations. J. DAIRY Sci. 100, 2119–2136. https://doi.org/10.3168/jds.2016-11517

Aldrin, M., Jansen, P.A., Stryhn, H., 2019. A partly stage-structured model for the abundance of salmon lice in salmonid farms. Epidemics 26, 9–22. https://doi.org/10.1016/j.epidem.2018.08.001

Al-Mouqatea, S., Alkhamis, M., Akbar, B., Ali, A., Al-Aqeel, H., Bin-Heji, A., Razzaque, M., Alvarez, J., Perez, A., 2018. Bayesian estimation of ELISA and gamma interferon test accuracy for the detection of bovine tuberculosis in caudal fold test-negative dairy cattle in Kuwait. J. Vet. Diagn. Investig. Off. Publ. Am. Assoc. Vet. Lab. Diagn. Inc 30, 468–470. https://doi.org/10.1177/1040638718759574

Amirpour Haredasht, S., Tavornpanich, S., Jansen, M.D., Lyngstad, T.M., Yatabe, T., Brun, E., Martínez-López, B., 2019. A stochastic network-based model to simulate the spread of pancreas disease (PD) in the Norwegian salmon industry based on the observed vessel movements and seaway distance between marine farms. Prev. Vet. Med. 167, 174–181. https://doi.org/10.1016/j.prevetmed.2018.05.019

Anstey, I., Quigley, B.L., Polkinghorne, A., Jelocnik, M., 2019. Chlamydial infection and on-farm risk factors in dairy cattle herds in South East Queensland. Aust. Vet. J. 97, 505–508. https://doi.org/10.1111/avj.12879

Arriagada, G., Hamilton-West, C., Nekouei, O., Foerster, C., Müller, A., Lara, M., Gallardo-Escárate, C., 2019. Caligus rogercresseyi infestation is associated with Piscirickettsia salmonis-attributed mortalities in farmed salmonids in Chile. Prev. Vet. Med. 171, 104771. https://doi.org/10.1016/j.prevetmed.2019.104771

Arriagada, G., Sanchez, J., Stryhn, H., Vanderstichel, R., Campistó, J.L., Ibarra, R., St-Hilaire, S., 2018. A multivariable assessment of the spatio-temporal distribution of pyrethroids performance on the sea lice Caligus rogercresseyi in Chile. Spat. Spatio-Temporal Epidemiol. 26, 1–13. https://doi.org/10.1016/j.sste.2018.04.004

Aslam, M.L., Robledo, D., Krasnov, A., Moghadam, H.K., Hillestad, B., Houston, R.D., Baranski, M., Boison, S., Robinson, N.A., 2020. Quantitative trait loci and genes associated with salmonid alphavirus load in Atlantic salmon: implications for pancreas disease resistance and tolerance. Sci. Rep. 10. https://doi.org/10.1038/s41598-020-67405-8

Bang Jensen, B., Nylund, S., Svendsen, J.C., Ski, P.-M.R., Takle, H., 2019. Indications for a vertical transmission pathway of piscine myocarditis virus in Atlantic salmon (Salmo salar L.). J. Fish Dis. 42, 825–833. https://doi.org/10.1111/jfd.12990

Bang Jensen, B., Qviller, L., Toft, N., 2020. Spatio-temporal variations in mortality during the seawater production phase of Atlantic salmon (Salmo salar) in Norway. J. Fish Dis. 43, 445–457. https://doi.org/10.1111/jfd.13142

Barker, S.E., Bricknell, I.R., Covello, J., Purcell, S., Fast, M.D., Wolters, W., Bouchard, D.A., 2019. Sea lice, Lepeophtheirus salmonis (Krøyer 1837), infected Atlantic salmon (Salmo salar L.) are more susceptible to infectious salmon anemia virus. PLOS ONE 14, e0209178. https://doi.org/10.1371/journal.pone.0209178

Bayliss, S.C., Verner-Jeffreys, D.W., Bartie, K.L., Aanensen, D.M., Sheppard, S.K., Adams, A., Feil, E.J., 2017. The Promise of Whole Genome Pathogen Sequencing for the Molecular Epidemiology

of Emerging Aquaculture Pathogens. Front. Microbiol. 8, 121. https://doi.org/10.3389/fmicb.2017.00121
Bayliss, S.C., Verner-Jeffreys, D.W., Ryder, D., Suarez, R., Ramirez, R., Romero, J., Pascoe, B., Sheppard, S.K., Godoy, M., Feil, E.J., 2018. Genomic epidemiology of the commercially important pathogen Renibacterium salmoninarum within the Chilean salmon industry. Microb. Genomics 4. https://doi.org/10.1099/mgen.0.000201
Beaver, A., Meagher, R.K., von Keyserlingk, M.A.G., Weary, D.M., 2019. Invited review: A systematic review of the effects of early separation on dairy cow and calf health. J. DAIRY Sci. 102, 5784–5810. https://doi.org/10.3168/jds.2018-15603
Biemans, F., Bijma, P., Boots, N.M., de Jong, M.C.M., 2018. Digital Dermatitis in dairy cattle: The contribution of different disease classes to transmission. Epidemics 23, 76–84. https://doi.org/10.1016/j.epidem.2017.12.007
Blandford, M.I., Taylor-Brown, A., Schlacher, T.A., Nowak, B., Polkinghorne, A., 2018. Epitheliocystis in fish: An emerging aquaculture disease with a global impact. Transbound. Emerg. Dis. 65, 1436–1446. https://doi.org/10.1111/tbed.12908
Boerlage, A.S., Ashby, A., Herrero, A., Reeves, A., Gunn, G.J., Rodger, H.D., 2020. Epidemiology of marine gill diseases in Atlantic salmon (Salmo salar) aquaculture: a review. Rev. Aquac. https://doi.org/10.1111/raq.12426
Boerlage, A.S., Elghafghuf, A., Stryhn, H., Sanchez, J., Hammell, K.L., 2018. Risk factors associated with time to first clinical case of Bacterial Kidney Disease (BKD) in farmed Atlantic Salmon (Salmo salar L.) in New Brunswick, Canada. Prev. Vet. Med. 149, 98–106. https://doi.org/10.1016/j.prevetmed.2017.11.014
Boerlage, A.S., Stryhn, H., Armstrong, B., Hammell, K.L., 2019. A 2-stage hierarchical interrupted time-series analysis to quantify the long-term effect of subclinical bacterial kidney disease on performance of farmed Atlantic salmon (Salmo salar L.). Prev. Vet. Med. 172, 104776. https://doi.org/10.1016/j.prevetmed.2019.104776
Boerlage, A.S., Stryhn, H., Sanchez, J., Hammell, K.L., 2017. Case definition for clinical and subclinical bacterial kidney disease (BKD) in Atlantic Salmon (Salmo salar L.) in New Brunswick, Canada. J. Fish Dis. 40, 395–409. https://doi.org/10.1111/jfd.12521
Borges, I.A., McCollum, A.M., Mehal, J.M., Haberling, D., Dutra, L.A.L., Vieira, F.N., Andrade, L.A.O., Kroon, E.G., Holman, R.C., Reynolds, M.G., Trindade, G.S., 2017. Dairy production practices and associated risks for bovine vaccinia exposure in cattle, Brazil. New Microbes New Infect. 20, 43–50. https://doi.org/10.1016/j.nmni.2017.08.004
Bravo, F., Sidhu, J.P.S., Bernal, P., Bustamante, R.H., Condie, S., Gorton, B., Herzfeld, M., Jimenez, D., Mardones, F.O., Rizwi, F., Steven, A.D.L., 2020. Hydrodynamic connectivity, water temperature, and salinity are major drivers of piscirickettsiosis prevalence and transmission among salmonid farms in Chile. Aquac. Environ. Interact. 12, 263–279. https://doi.org/10.3354/aei00368
Brosnahan, C.L., Munday, J.S., Ha, H.J., Preece, M., Jones, J.B., 2019. New Zealand rickettsia-like organism (NZ-RLO) and Tenacibaculum maritimum: Distribution and phylogeny in farmed Chinook salmon (Oncorhynchus tshawytscha). J. Fish Dis. 42, 85–95. https://doi.org/10.1111/jfd.12909
Buczinski, S., Borris, M.E., Dubuc, J., 2018. Herd-level prevalence of the ultrasonographic lung lesions associated with bovine respiratory disease and related environmental risk factors. J. DAIRY Sci. 101, 2423–2432. https://doi.org/10.3168/jds.2017-13459
Büyükekiz, A.G., Altun, S., Hansen, E.F., Satıcıoğlu, I.B., Duman, M., Markussen, T., Rimstad, E., 2018. Infectious pancreatic necrosis virus (IPNV) serotype Sp is prevalent in Turkish rainbow trout farms. J. Fish Dis. 41, 95–104. https://doi.org/10.1111/jfd.12675
Chatziprodromidou, I.P., Apostolou, T., 2018. Diagnostic accuracy of enzyme-linked immunosorbent assay (ELISA) and immunoblot (IB) for the detection of antibodies against Neospora caninum

in milk from dairy cows. Epidemiol. Infect. 146, 577–583. https://doi.org/10.1017/S0950268817002916
Chung, J.J., Rayburn, M.C., Chigerwe, M., 2019. Randomized controlled clinical trial on the effect of oral immunoglobulin supplementation on neonatal dairy calves with diarrhea. J. Vet. Intern. Med. 33, 1807–1813. https://doi.org/10.1111/jvim.15538
Corbett, C.S., de Jong, M.C.M., Orsel, K., De Buck, J., Barkema, H.W., 2019. Quantifying transmission of Mycobacterium avium subsp. paratuberculosis among group-housed dairy calves. Vet. Res. 50, 60. https://doi.org/10.1186/s13567-019-0678-3
Cruvinel, L.B., Nicaretta, J.E., Bastos, T.S.A., Couto, L.F.M., Dos Santos, J.B., Zapa, D.M.B., Cavalcante, A.S.A., Cruz, B.C., Borges, D.G.L., Borges, F.A., Soares, V.E., Lopes, W.D.Z., 2018. Eimeria species in dairy and beef cattle of different ages in Goiás state, Brazil. Rev. Bras. Parasitol. Vet. 27, 169–176. https://doi.org/10.1590/S1984-296120180038
Cummings, K.J., Virkler, P.D., Wagner, B., Lussier, E.A., Thompson, B.S., 2018. Herd-level prevalence of Salmonella Dublin among New York dairy farms based on antibody testing of bulk tank milk. ZOONOSES PUBLIC Health 65, 1003–1007. https://doi.org/10.1111/zph.12523
Cvetojevic, D., Milicevic, V., Kureljusic, B., Savic, B., 2018. Seroprevalence of Neospora caninum in dairy cows in Belgrade city area, Serbia. J. Hell. Vet. Med. Soc. 69, 979–983. https://doi.org/10.12681/jhvms.18026
Dalen, G., Rachah, A., Nørstebø, H., Schukken, Y.H., Reksen, O., 2019. Dynamics of somatic cell count patterns as a proxy for transmission of mastitis pathogens. J. DAIRY Sci. 102, 11349–11358. https://doi.org/10.3168/jds.2019-16847
Daros, R.R., Eriksson, H.K., Weary, D.M., von Keyserlingk, M.A.G., 2020. The relationship between transition period diseases and lameness, feeding time, and body condition during the dry period. J. DAIRY Sci. 103, 649–665. https://doi.org/10.3168/jds.2019-16975
Deikun, L.L., Habing, G.G., Quigley, J.D., Proudfoot, K.L., 2020. Health and growth of veal calves provided a fatty acid supplement and a dry teat. J. DAIRY Sci. 103, 4633–4642. https://doi.org/10.3168/jds.2019-17240
Deperasinska, I., Schulz, P., Siwicki, A.K., 2018. Salmonid alphavirus (SAV). J. Vet. Res. 62, 1–6. https://doi.org/10.2478/jvetres-2018-0001
Dessen, J.-E., Mørkøre, T., Bildøy, J.I., Johnsen, S.N., Poppe, L.T., Hatlen, B., Thomassen, M.S., Rørvik, K.-A., 2019. Increased dietary protein-to-lipid ratio improves survival during naturally occurring pancreas disease in Atlantic salmon, Salmo salar L. J. Fish Dis. 42, 21–34. https://doi.org/10.1111/jfd.12904
Dolecheck, K.A., Overton, M.W., Mark, T.B., Bewley, J.M., 2019. Use of a stochastic simulation model to estimate the cost per case of digital dermatitis, sole ulcer, and white line disease by parity group and incidence timing. J. DAIRY Sci. 102, 715–730. https://doi.org/10.3168/jds.2018-14901
Douphrate, D.I., Fethke, N.B., Nonnenmann, M.W., Rodriguez, A., Gimeno Ruiz de Porras, D., 2019. Reliability of observational- and machine-based teat hygiene scoring methodologies. J. DAIRY Sci. 102, 7494–7502. https://doi.org/10.3168/jds.2019-16351
Du, X., Bayliss, S.C., Feil, E.J., Liu, Y., Wang, C., Zhang, G., Zhou, D., Wei, D., Tang, N., Leclercq, S.O., Feng, J., 2019. Real time monitoring of Aeromonas salmonicida evolution in response to successive antibiotic therapies in a commercial fish farm. Environ. Microbiol. 21, 1113–1123. https://doi.org/10.1111/1462-2920.14531
Dubuc, J., 2017. Short communication: Diagnostic performance of on-farm bacteriological culture systems for identification of uterine Escherichia coli in postpartum dairy cows. J. DAIRY Sci. 100, 3079–3082. https://doi.org/10.3168/jds.2016-12315
Duman, M., Altun, S., Cengiz, M., Saticioglu, I.B., Buyukekiz, A.G., Sahinturk, P., 2017. Genotyping and antimicrobial resistance genes of Yersinia ruckeri isolates from rainbow trout farms. Dis. Aquat. Organ. 125, 31–44. https://doi.org/10.3354/dao03132

Duman, M., Buyukekiz, A.G., Saticioglu, I.B., Cengiz, M., Sahinturk, P., Altun, S., 2020. Epidemiology, genotypic diversity, and antimicrobial resistance of Lactococcus garvieae in farmed rainbow trout (Oncorhynchus mykiss). Iran. J. Fish. Sci. 19, 1–18. https://doi.org/10.22092/ijfs.2018.117609

Ekman, L., Nyman, A.-K., Landin, H., Magnusson, U., Waller, K.P., 2018a. Mild and severe udder cleft dermatitis-Prevalence and risk factors in Swedish dairy herds. J. DAIRY Sci. 101, 556–571. https://doi.org/10.3168/jds.2017-13133

Ekman, L., Nyman, A.-K., Landin, H., Persson Waller, K., 2018b. Hock lesions in dairy cows in freestall herds: A cross-sectional study of prevalence and risk factors. ACTA Vet. Scand. 60. https://doi.org/10.1186/s13028-018-0401-9

Ellis, J., DeLong, K.L., Lambert, D.M., Schexnayder, S., Krawczel, P., Oliver, S., 2020. Analysis of closed versus operating dairies in the southeastern United States. J. DAIRY Sci. 103, 5148–5161. https://doi.org/10.3168/jds.2019-17516

Escobar, L.E., Escobar-Dodero, J., Phelps, N.B.D., 2018. Infectious disease in fish: global risk of viral hemorrhagic septicemia virus. Rev. Fish Biol. Fish. 28, 637–655. https://doi.org/10.1007/s11160-018-9524-3

Escobar-Dodero, J., Kinsley, A., Perez, A.M., Ibarra, R., Tello, A., Monti, G., Mardones, F.O., 2019. Risk factors for infectious pancreatic necrosis in farmed Chilean Atlantic salmon (Salmo salar L.) from 2010 to 2013. Prev. Vet. Med. 167, 182–189. https://doi.org/10.1016/j.prevetmed.2018.04.016

Esmaeili, S., Mohabati Mobarez, A., Khalili, M., Mostafavi, E., 2019. High prevalence and risk factors of Coxiella burnetii in milk of dairy animals with a history of abortion in Iran. Comp. Immunol. Microbiol. Infect. Dis. 63, 127–130. https://doi.org/10.1016/j.cimid.2019.01.015

Esser, N.M., Su, H., Coblentz, W.K., Akins, M.S., Kieke, B.A., Martin, N.P., Borchardt, M.A., Jokela, W.E., 2019. Efficacy of recycled sand or organic solids as bedding sources for lactating cows housed in freestalls. J. DAIRY Sci. 102, 6682–6698. https://doi.org/10.3168/jds.2018-15851

Estevez, R.A., Mardones, F.O., Alamos, F., Arriagada, G., Carey, J., Corre, C., Escobar-Dodero, J., Gaete, A., Gallardo, A., Ibarra, R., Ortiz, C., Rozas-Serri, M., Sandoval, O., Santana, J., Gelcich, S., 2019. Eliciting expert judgements to estimate risk and protective factors for Piscirickettsiosis in Chilean salmon farming. AQUACULTURE 507, 402–410. https://doi.org/10.1016/j.aquaculture.2019.04.028

Faisal, M., Loch, T.P., Shavalier, M., VanDeuren, M.G., Standish, I., Winters, A., Glenney, G., Aho, J., Wolgamood, M., VanAmberg, J., Eisch, E., Whelan, G.E., 2019. Resurgence of Salmonid Herpesvirus-3 Infection (Epizootic Epitheliotropic Disease) in Hatchery-Propagated Lake Trout in Michigan. J. Aquat. Anim. Health 31, 31–45. https://doi.org/10.1002/aah.10051

Feist, S.W., Thrush, M.A., Dunn, P., Bateman, K., Peeler, E.J., 2019. The aquatic animal pandemic crisis. Rev. Sci. Tech. Int. Off. Epizoot. 38, 437–457. https://doi.org/10.20506/rst.38.2.2997

Fraslin, C., Brard-Fudulea, S., D'Ambrosio, J., Bestin, A., Charles, M., Haffray, P., Quillet, E., Phocas, F., 2019. Rainbow trout resistance to bacterial cold water disease: two new quantitative trait loci identified after a natural disease outbreak on a French farm. Anim. Genet. 50, 293–297. https://doi.org/10.1111/age.12777

Gaete-Carrasco, A., Rosenfeld, C., Gallardo, A., 2019. Epidemiological analysis of the active surveillance programme for Piscirickettsia salmonis of the National Fisheries and Aquaculture Service of Chile. Rev. Sci. Tech. Int. Off. Epizoot. 38, 823–849. https://doi.org/10.20506/rst.38.3.3029

Gallagher, M.D., Karlsen, M., Petterson, E., Haugland, O., Matejusova, I., Macqueen, D.J., 2020. Genome sequencing of SAV3 reveals repeated seeding events of viral strains in norwegian aquaculture. Front. Microbiol. 11. https://doi.org/10.3389/fmicb.2020.00740

Gao, Y., Cao, J., Zhang, S., Zhang, Q., Sun, D., 2018. Short communication: Heritability estimates for susceptibility to Mycobacterium avium ssp. paratuberculosis infection in Chinese Holstein cattle. J. DAIRY Sci. 101, 7274–7279. https://doi.org/10.3168/jds.2017-13264

Garseth, Å.H., Fritsvold, C., Svendsen, J.C., Bang Jensen, B., Mikalsen, A.B., 2018. Cardiomyopathy syndrome in Atlantic salmon Salmo salar L.: A review of the current state of knowledge. J. Fish Dis. 41, 11–26. https://doi.org/10.1111/jfd.12735
Gautam, R., Price, D., Revie, C.W., Gardner, I.A., Vanderstichel, R., Gustafson, L., Klotins, K., Beattie, M., 2018. Connectivity-based risk ranking of infectious salmon anaemia virus (ISAv) outbreaks for targeted surveillance planning in Canada and the USA. Prev. Vet. Med. 159, 92–98. https://doi.org/10.1016/j.prevetmed.2018.09.001
Gautam, R., Vanderstichel, R., Boerlage, A.S., Revie, C.W., Hammell, K.L., 2017a. Effect of timing of count events on estimates of sea lice abundance and interpretation of effectiveness following bath treatments. J. Fish Dis. 40, 367–375. https://doi.org/10.1111/jfd.12519
Gautam, R., Vanderstichel, R., Boerlage, A.S., Revie, C.W., Hammell, K.L., 2017b. Evaluating bath treatment effectiveness in the control of sea lice burdens on Atlantic salmon in New Brunswick, Canada. J. Fish Dis. 40, 895–905. https://doi.org/10.1111/jfd.12569
Gislason, H., 2018. Statistical modelling of sea lice count data from salmon farms in the Faroe Islands. J. Fish Dis. 41, 973–993. https://doi.org/10.1111/jfd.12742
Gohary, K., LeBlanc, S.J., 2018. Cost of retained fetal membranes for dairy herds in the United States. JAVMA-J. Am. Vet. Med. Assoc. 252, 1485–1489. https://doi.org/doi.org/10.2460/javma.252.12.1485
Gomez, D.E., Arroyo, L.G., Poljak, Z., Viel, L., Weese, J.S., 2017. Detection of bovine coronavirus in healthy and diarrheic dairy calves. J. Vet. Intern. Med. 31, 1884–1891. https://doi.org/10.1111/jvim.14811
Goto, A., Takahara, K., Sugiura, T., Oikawa, S., Katamato, H., Nakada, K., 2019. Association of postpartum diseases occurring within 60 days after calving with productivity and reproductive performance in dairy cows in Fukuoka: A cow-level, retrospective cohort study. J. Vet. Med. Sci. 81, 1055–1062. https://doi.org/10.1292/jvms.18-0384
Guarracino, M., Qviller, L., Lillehaug, A., 2018. Evaluation of aquaculture management zones as a control measure for salmon lice in Norway. Dis. Aquat. Organ. 130, 1–9. https://doi.org/10.3354/dao03254
Guerrero-Cabrera, L., Luna Olivera, B.C., Villavicencio-Pulido, J.G., Ortiz Luna, R.J., 2020. Proximity and density of neighboring farms and water supply, as risk factors for bacteriosis: A case study of spatial risk analysis in tilapia and rainbow trout farms of Oaxaca, Mexico. Aquaculture 520, 734955. https://doi.org/10.1016/j.aquaculture.2020.734955
Guimarães, F.F., Manzi, M.P., Joaquim, S.F., Richini-Pereira, V.B., Langoni, H., 2017. Short communication: Outbreak of methicillin-resistant Staphylococcus aureus (MRSA)-associated mastitis in a closed dairy herd. J. DAIRY Sci. 100, 726–730. https://doi.org/10.3168/jds.2016-11700
Gussmann, M., Steeneveld, W., Kirkeby, C., Hogeveen, H., Farre, M., Halasa, T., 2019. Economic and epidemiological impact of different intervention strategies for subclinical and clinical mastitis. Prev. Vet. Med. 166, 78–85. https://doi.org/10.1016/j.prevetmed.2019.03.001
Haine, D., Delgado, H., Cue, R., Sewalem, A., Wade, K., Lacroix, R., Lefebvre, D., Arsenault, J., Bouchard, É., Dubuc, J., 2017. Culling from the herd's perspective—Exploring herd-level management factors and culling rates in Québec dairy herds. Prev. Vet. Med. 147, 132–141. https://doi.org/10.1016/j.prevetmed.2017.08.020
Happold, J., Meyer, A., Sadler, R., Cowled, B., Mackenzie, C., Gallardo Lagno, A., Cameron, A., 2020a. Effectiveness of antimicrobial treatment of salmonid rickettsial septicaemia in commercial salmon and trout farms in Chile. Aquaculture 525, 735323. https://doi.org/doi.org/10.1016/j.aquaculture.2020.735323
Happold, J., Sadler, R., Meyer, A., Cowled, B., Mackenzie, C., Gallardo Lagno, A., Cameron, A., 2020b. Effectiveness of vaccination for the control of salmonid rickettsial septicaemia in commercial salmon and trout farms in Chile. Aquaculture 520, 734968. https://doi.org/10.1016/j.aquaculture.2020.734968

Hayer, S.S., VanderWaal, K., Ranjan, R., Biswal, J.K., Subramaniam, S., Mohapatra, J.K., Sharma, G.K., Rout, M., Dash, B.B., Das, B., Prusty, B.R., Sharma, A.K., Stenfeldt, C., Perez, A., Delgado, A.H., Sharma, M.K., Rodriguez, L.L., Pattnaik, B., Arzt, J., 2018. Foot-and-mouth disease virus transmission dynamics and persistence in a herd of vaccinated dairy cattle in India. Transbound. Emerg. Dis. 65, e404–e415. https://doi.org/10.1111/tbed.12774

Hellebø, A., Stene, A., Aspehaug, V., 2017. PCR survey for Paramoeba perurans in fauna, environmental samples and fish associated with marine farming sites for Atlantic salmon (Salmo salar L.). J. Fish Dis. 40, 661–670. https://doi.org/10.1111/jfd.12546

Hillman, A., Zalcman, E., Burroughs, A., Meyer, A., Mackenzie, C., Sadler, R., Stevenson, M., Ward, M., Gallardo Lagno, A., Happold, J., Cameron, A., Cowled, B., 2020. Use of regulatory data to describe and identify risk factors for salmonid rickettsial septicaemia in Chile, 2011–2017. Aquaculture 526, 735324. https://doi.org/doi.org/10.1016/j.aquaculture.2020.735324

Isla, A., Saldarriaga-Córdoba, M., Fuentes, D.E., Albornoz, R., Haussmann, D., Mancilla-Schulz, J., Martínez, A., Figueroa, J., Avendaño-Herrera, R., Yáñez, A., 2019. Multilocus sequence typing detects new Piscirickettsia salmonis hybrid genogroup in Chilean fish farms: Evidence for genetic diversity and population structure. J. Fish Dis. 42, 721–737. https://doi.org/10.1111/jfd.12976

Jagielski, T., Krukowski, H., Bochniarz, M., Piech, T., Roeske, K., Bakuła, Z., Wlazło, Ł., Woch, P., 2019. Prevalence of Prototheca spp. on dairy farms in Poland - a cross-country study. Microb. Biotechnol. 12, 556–566. https://doi.org/10.1111/1751-7915.13394

Jalali, S.A.H., Mohammadinezhad, R., Mohammadi, A., Latifian, M.H., Talebi, M., Soleimanin-Zad, S., Golkar, P., Hemmatzadeh, F., 2019. Molecular evolution and selection pressure analysis of infectious hematopoietic necrosis virus (IHNV) revealed the origin and phylogenetic relationship of Iranian isolates in recent epidemics in Iran. Virology 535, 45–58. https://doi.org/10.1016/j.virol.2019.06.012

Jamali, H., Barkema, H.W., Jacques, M., Lavallée-Bourget, E.-M., Malouin, F., Saini, V., Stryhn, H., Dufour, S., 2018. Invited review: Incidence, risk factors, and effects of clinical mastitis recurrence in dairy cows. J. DAIRY Sci. 101, 4729–4746. https://doi.org/10.3168/jds.2017-13730

Jansen, M.D., Bang Jensen, B., McLoughlin, M.F., Rodger, H.D., Taksdal, T., Sindre, H., Graham, D.A., Lillehaug, A., 2017. The epidemiology of pancreas disease in salmonid aquaculture: a summary of the current state of knowledge. J. Fish Dis. 40, 141–155. https://doi.org/10.1111/jfd.12478

Jansen, M.D., Guarracino, M., Carson, M., Modahl, I., Taksdal, T., Sindre, H., Brun, E., Tavornpanich, S., 2019. Field evaluation of diagnostic test sensitivity and specificity for salmonid alphavirus (SAV) infection and pancreas disease (PD) in farmed atlantic salmon (salmo salar l.) in norway using bayesian latent class analysis. Front. Vet. Sci. 6. https://doi.org/10.3389/fvets.2019.00419

Janssen, K., Komen, H., Saatkamp, H.W., de Jong, M.C.M., Bijma, P., 2018. Derivation of the economic value of R(0) for macroparasitic diseases and application to sea lice in salmon. Genet. Sel. Evol. GSE 50, 47. https://doi.org/10.1186/s12711-018-0418-6

Jia, P., Breyta, R.B., Li, Q., Qian, X., Wu, B., Zheng, W., Wen, Z., Liu, Y., Kurath, G., Hua, Q., Jin, N., Liu, H., 2018. Insight into infectious hematopoietic necrosis virus (IHNV) in Chinese rainbow trout aquaculture from virus isolated from 7 provinces in 2010–2014. Aquaculture 496, 239–246. https://doi.org/10.1016/j.aquaculture.2018.06.062

Johnsen, I.A., Stien, L.H., Sandvik, A.D., Asplin, L., Oppedal, F., 2020. Optimal estimation of lice release from aquaculture based on ambient temperatures. Aquac. Environ. Interact. 12, 179–191. https://doi.org/10.3354/aei00358

Johnson-Mackinnon, J.C., Crosbie, P.B.B., Karlsbakk, E., Marcos-Lopez, M., Paley, R., Nowak, B.F., Bridle, A.R., 2019. Multilocus Sequence Typing (MLST) and Random Polymorphic DNA (RAPD)

Comparisons of Geographic Isolates of Neoparamoeba perurans, the Causative Agent of Amoebic Gill Disease. Pathog. Basel Switz. 8. https://doi.org/10.3390/pathogens8040244
Jones, A.E., Munro, L.A., Green, D.M., Morgan, K.L., Murray, A.G., Norman, R., Ryder, D., Salama, N.K.G., Taylor, N.G.H., Thrush, M.A., Wallace, I.S., Sharkey, K.J., 2019. The contact structure of Great Britain's salmon and trout aquaculture industry. Epidemics 28, 100342. https://doi.org/10.1016/j.epidem.2019.05.001
Jones, S.R.M., Long, A., MacWilliams, C., Polinski, M., Garver, K., 2020. Factors associated with severity of naturally occurring piscirickettsiosis in netpen- and tank-reared juvenile Atlantic salmon at a research aquarium in western Canada. J. Fish Dis. 43, 49–55. https://doi.org/10.1111/jfd.13102
Karami, A.M., Bani, A., Pourkazemi, M., Ghasemi, M., Kania, P.W., Buchmann, K., 2018. Comparative susceptibilities and immune reactions of wild and cultured populations of Caspian trout Salmo trutta caspius to VHSV. Dis. Aquat. Organ. 128, 187–201. https://doi.org/10.3354/dao03231
Karlsen, C., Ottem, K.F., Brevik, Ø.J., Davey, M., Sørum, H., Winther-Larsen, H.C., 2017. The environmental and host-associated bacterial microbiota of Arctic seawater-farmed Atlantic salmon with ulcerative disorders. J. Fish Dis. 40, 1645–1663. https://doi.org/10.1111/jfd.12632
Karzis, J., Petzer, I.-M., Donkin, E.F., Naidoo, V., 2018. Proactive udder health management in South Africa and monitoring of antibiotic resistance of Staphylococcus aureus; in dairy herds from 2001 to 2010. J. S. Afr. Vet. Assoc. 89, e1–e8. https://doi.org/10.4102/jsava.v89i0.1490
Kelley, J.M., Rathinasamy, V., Elliott, T.P., Rawlin, G., Beddoe, T., Stevenson, M.A., Spithill, T.W., 2020. Determination of the prevalence and intensity of Fasciola hepatica infection in dairy cattle from six irrigation regions of Victoria, South-eastern Australia, further identifying significant triclabendazole resistance on three properties. Vet. Parasitol. 277, 109019. https://doi.org/10.1016/j.vetpar.2019.109019
Kelly, E., McAloon, C.G., O'Grady, L., Duane, M., Somers, J.R., Beltman, M.E., 2020. Cow-level risk factors for reproductive tract disease diagnosed by 2 methods in pasture-grazed dairy cattle in Ireland. J. DAIRY Sci. 103, 737–749. https://doi.org/10.3168/jds.2019-17064
Kibenge, M.J.T., Wang, Y., Gayeski, N., Morton, A., Beardslee, K., McMillan, B., Kibenge, F.S.B., 2019. Piscine orthoreovirus sequences in escaped farmed Atlantic salmon in Washington and British Columbia. Virol. J. 16, 41. https://doi.org/10.1186/s12985-019-1148-2
Knupp, C., Wiens, G.D., Faisal, M., Call, D.R., Cain, K.D., Nicolas, P., Van Vliet, D., Yamashita, C., Ferguson, J.A., Meuninck, D., Hsu, H.-M., Baker, B.B., Shen, L., Loch, T.P., 2019. Large-Scale Analysis of Flavobacterium psychrophilum Multilocus Sequence Typing Genotypes Recovered from North American Salmonids Indicates that both Newly Identified and Recurrent Clonal Complexes Are Associated with Disease. Appl. Environ. Microbiol. 85. https://doi.org/10.1128/AEM.02305-18
Kristoffersen, A.B., Devold, M., Aspehaug, V., Gjelstenli, O., Breck, O., Bang Jensen, B., 2018. Molecular tracing confirms that infection with infectious pancreatic necrosis virus follows the smolt from hatchery to grow-out farm. J. Fish Dis. 41, 1601–1607. https://doi.org/10.1111/jfd.12844
la Bastide, P.Y. de, Naumann, C., Hintz, W.E., 2018. Assessment of intra-specific variability in Saprolegnia parasitica populations of aquaculture facilities in British Columbia, Canada. Dis. Aquat. Organ. 128, 235–248. https://doi.org/10.3354/dao03224
Lillehaug, A., Børnes, C., Grave, K., 2018. A pharmaco-epidemiological study of antibacterial treatments and bacterial diseases in Norwegian aquaculture from 2011 to 2016. Dis. Aquat. Organ. 128, 117–125. https://doi.org/10.3354/dao03219
Lipkens, Z., Piepers, S., De Visscher, A., De Vliegher, S., 2019. Evaluation of test-day milk somatic cell count information to predict intramammary infection with major pathogens in dairy cattle at drying off. J. DAIRY Sci. 102, 4309–4321. https://doi.org/10.3168/jds.2018-15642

Liu, H., Li, S., Meng, L., Dong, L., Zhao, S., Lan, X., Wang, J., Zheng, N., 2017. Prevalence, antimicrobial susceptibility, and molecular characterization of Staphylococcus aureus isolated from dairy herds in northern China. J. DAIRY Sci. 100, 8796–8803. https://doi.org/10.3168/jds.2017-13370

Loch, T.P., Faisal, M., 2018. Flavobacteria colonizing the early life stages of hatchery-incubated Chinook salmon Oncorhynchus tshawytscha (Walbaum 1792) are markedly diverse. J. Fish Dis. 41, 829–845. https://doi.org/10.1111/jfd.12795

Macchi, M.V., Suanes, A., Salaberry, X., Fernandez, F., Piaggio, J., Gil, A.D., 2020. Epidemiological study of neosporosis in Uruguayan dairy herds. Prev. Vet. Med. 179. https://doi.org/10.1016/j.prevetmed.2020.105022

MacKinnon, B., Jones, P., Hawkins, L., Dohoo, I., Stryhn, H., Vanderstichel, R., St-Hilaire, S., 2019. The epidemiology of skin ulcers in saltwater reared Atlantic salmon (Salmo salar) in Atlantic Canada. Aquaculture 501, 230–238. https://doi.org/10.1016/j.aquaculture.2018.11.035

Mahendran, S.A., Booth, R., Beekhuis, L., Manning, A., Blackmore, T., Vanhoudt, A., Bell, N., 2017. Assessing the effects of weekly preweaning health scores on dairy calf mortality and productivity parameters: cohort study. Vet. Rec. 181, 196. https://doi.org/10.1136/vr.104197

Mancilla-Schulz, J., Marín, S.L., Molinet, C., 2019. Dynamics of Caligus rogercresseyi (Boxshall & Bravo, 2000) in farmed Atlantic salmon (Salmo salar) in southern Chile: Are we controlling sea lice? J. Fish Dis. 42, 357–369. https://doi.org/10.1111/jfd.12931

Mardones, F.O., Paredes, F., Medina, M., Tello, A., Valdivia, V., Ibarra, R., Correa, J., Gelcich, S., 2018. Identification of research gaps for highly infectious diseases in aquaculture: The case of the endemic Piscirickettsia salmonis in the Chilean salmon farming industry. Aquaculture 482, 211–220. https://doi.org/10.1016/j.aquaculture.2017.09.048

Medeiros, T.N.S., Lorenzetti, E., Massi, R.P., Alfieri, A.F., Alfieri, A.A., 2020. Neonatal diarrhea and rotavirus A infection in beef and dairy calves, Brazil, 2006-2015 [Diarreia neonatal e infecção por rotavírus A em bezerros de corte e leite, Brasil, 2006-2015]. Pesqui. Vet. Bras. 40, 7–11. https://doi.org/10.1590/1678-5150-PVB-5919

Meyer, A, Burroughs, A, Sadler, R, Happold, J, Cowled, B, Mackenzie, C., Gallardo Lagno, AL, Cameron, A., 2019. Quantifying the effects of sea lice burden and lice bathing treatments on salmonid rickettsial septicaemia in commercial salmon and trout farms in Chile. Aquaculture 513, 734411. https://doi.org/10.1016/j.aquaculture.2019.734411

Meyer, A., Sadler, R., Bannister-Tyrrell, M., Gallardo Lagno, AL, Stegeman, A., Cameron, A., 2020. Is between-farm water-borne pathogen dissemination an important driver in the epidemiology of salmonid rickettsial septicaemia in Chile? Aquaculture 530, 735751. https://doi.org/10.1016/j.aquaculture.2020.735751

Milne, M.G., Graham, J., Allen, A., McCormick, C., Presho, E., Skuce, R., Byrne, A.W., 2019. Variation in Mycobacterium bovis genetic richness suggests that inwards cattle movements are a more important source of infection in beef herds than in dairy herds. BMC Microbiol. 19. https://doi.org/10.1186/s12866-019-1530-7

Miranda, C.D., Godoy, F.A., Lee, M.R., 2018. Current Status of the Use of Antibiotics and the Antimicrobial Resistance in the Chilean Salmon Farms. Front. Microbiol. 9, 1284. https://doi.org/10.3389/fmicb.2018.01284

Miyama, T., Byaruhanga, J., Okamura, I., Nagahata, H., Murata, R., Mwebembezi, W., Muramatsu, Y., Makita, K., 2020. Prevalence of sub-clinical mastitis and its association with milking practices in an intensive dairy production region of Uganda. J. Vet. Med. Sci. 82, 488–493. https://doi.org/10.1292/jvms.19-0588

Mullin, B.R., Reyda, F.B., 2020. High Prevalence of the Copepod Salmincola californiensis in Steelhead Trout in Lake Ontario Following its Recent Invasion. J. Parasitol. 106, 198–200.

Myksvoll, M.S., Sandvik, A.D., Albretsen, J., Asplin, L., Johnsen, I.A., Karlsen, Ø., Kristensen, N.M., Melsom, A., Skardhamar, J., Ådlandsvik, B., 2018. Evaluation of a national operational

salmon lice monitoring system-From physics to fish. PloS One 13, e0201338. https://doi.org/10.1371/journal.pone.0201338
Nekouei, O., Vanderstichel, R., Kaukinen, K.H., Thakur, K., Ming, T., Patterson, D.A., Trudel, M., Neville, C., Miller, K.M., 2019. Comparison of infectious agents detected from hatchery and wild juvenile Coho salmon in British Columbia, 2008-2018. PloS One 14, e0221956. https://doi.org/10.1371/journal.pone.0221956
Ngo, T.P.H., Bartie, K.L., Thompson, K.D., Verner-Jeffreys, D.W., Hoare, R., Adams, A., 2017. Genetic and serological diversity of Flavobacterium psychrophilum isolates from salmonids in United Kingdom. Vet. Microbiol. 201, 216–224. https://doi.org/10.1016/j.vetmic.2017.01.032
Olsen, A.B., Gulla, S., Steinum, T., Colquhoun, D.J., Nilsen, H.K., Duchaud, E., 2017. Multilocus sequence analysis reveals extensive genetic variety within Tenacibaculum spp. associated with ulcers in sea-farmed fish in Norway. Vet. Microbiol. 205, 39–45. https://doi.org/10.1016/j.vetmic.2017.04.028
Otten, N.D., Toft, N., Thomsen, P.T., Houe, H., 2019. Evaluation of the performance of register data as indicators for dairy herds with high lameness prevalence. ACTA Vet. Scand. 61, 49. https://doi.org/10.1186/s13028-019-0484-y
Pajdak-Czaus, J., Platt-Samoraj, A., Szweda, W., Siwicki, A.K., Terech-Majewska, E., 2019. Yersinia ruckeri—A threat not only to rainbow trout. Aquac. Res. 50, 3083–3096. https://doi.org/10.1111/are.14274
Pantoja, J.C.F., Correia, L.B.N., Rossi, R.S., Latosinski, G.S., 2020. Association between teat-end hyperkeratosis and mastitis in dairy cows: A systematic review. J. DAIRY Sci. 103, 1843–1855. https://doi.org/10.3168/jds.2019-16811
Park, H.-S., Moon, D.C., Hyun, B.-H., Lim, S.-K., 2019. Short communication: Occurrence and persistence of Prototheca zopfii in dairy herds of Korea. J. DAIRY Sci. 102, 2539–2543. https://doi.org/10.3168/jds.2018-14979
Peruzzi, S., Puvanendran, V., Riesen, G., Seim, R.R., Hagen, Ø., Martínez-Llorens, S., Falk-Petersen, I.-B., Fernandes, J.M.O., Jobling, M., 2018. Growth and development of skeletal anomalies in diploid and triploid Atlantic salmon (Salmo salar) fed phosphorus-rich diets with fish meal and hydrolyzed fish protein. PloS One 13, e0194340. https://doi.org/10.1371/journal.pone.0194340
Petersen, M.B., Wawegama, N.K., Denwood, M., Markham, P.F., Browning, G.F., Nielsen, L.R., 2018. Mycoplasma bovis antibody dynamics in naturally exposed dairy calves according to two diagnostic tests. BMC Vet. Res. 14. https://doi.org/10.1186/s12917-018-1574-1
Price, D., Ibarra, R., Sánchez, J., St-Hilaire, S., 2017. A retrospective assessment of the effect of fallowing on piscirickettsiosis in Chile. Aquaculture 473, 400–406. https://doi.org/10.1016/j.aquaculture.2017.02.034
Räihä, V., Sundberg, L.-R., Ashrafi, R., Hyvärinen, P., Karvonen, A., 2019. Rearing background and exposure environment together explain higher survival of aquaculture fish during a bacterial outbreak. J. Appl. Ecol. 56, 1741–1750. https://doi.org/10.1111/1365-2664.13393
Reimus, K., Orro, T., Emanuelson, U., Viltrop, A., Mõtus, K., 2018. On-farm mortality and related risk factors in Estonian dairy cows. Prev. Vet. Med. 155, 53–60. https://doi.org/10.1016/j.prevetmed.2018.04.006
Rodríguez, F.H., Flores-Mara, R., Yoshida, G.M., Barría, A., Jedlicki, A.M., Lhorente, J.P., Reyes-López, F., Yáñez, J.M., 2019. Genome-Wide Association Analysis for Resistance to Infectious Pancreatic Necrosis Virus Identifies Candidate Genes Involved in Viral Replication and Immune Response in Rainbow Trout (Oncorhynchus mykiss). G3 Bethesda Md 9, 2897–2904. https://doi.org/10.1534/g3.119.400463
Røsæg, M.V., Garseth, Å.H., Brynildsrud, O.B., Jansen, M.D., 2019. Pancreas disease caused by Salmonid alphavirus subtype 2 reduces growth and feed conversion in farmed Atlantic salmon. Prev. Vet. Med. 169, 104699. https://doi.org/10.1016/j.prevetmed.2019.104699

Rosaeg, M.V., Rimstad, E., Guttvik, A., Skjelstad, B., Bendiksen, E.A., Garseth, A.H., 2019. Effect of pancreas disease caused by SAV 2 on protein and fat digestion in Atlantic salmon. J. FISH Dis. 42, 97–108. https://doi.org/10.1111/jfd.12914
Rypuła, K., Płoneczka-Janeczko, K., Czopowicz, M., Klimowicz-Bodys, M.D., Shabunin, S., Siegwalt, G., 2020. Occurrence of BVDV Infection and the Presence of Potential Risk Factors in Dairy Cattle Herds in Poland. Anim. Open Access J. MDPI 10. https://doi.org/10.3390/ani10020230
Salama, N.K.G., Dale, A.C., Ivanov, V.V., Cook, P.F., Pert, C.C., Collins, C.M., Rabe, B., 2018. Using biological-physical modelling for informing sea lice dispersal in Loch Linnhe, Scotland. J. Fish Dis. 41, 901–919. https://doi.org/10.1111/jfd.12693
Samsing, F., Johnsen, I., Dempster, T., Oppedal, F., Treml, E.A., 2017. Network analysis reveals strong seasonality in the dispersal of a marine parasite and identifies areas for coordinated management. Landsc. Ecol. 32, 1953–1967. https://doi.org/10.1007/s10980-017-0557-0
Samsing, F., Johnsen, I., Treml, E.A., Dempster, T., 2019. Identifying “firebreaks” to fragment dispersal networks of a marine parasite. Int. J. Parasitol. 49, 277–286. https://doi.org/10.1016/j.ijpara.2018.11.005
Scott, H., Gilleard, J.S., Jelinski, M., Barkema, H.W., Redman, E.M., Avramenko, R.W., Luby, C., Kelton, D.F., Bauman, C.A., Keefe, G., Dubuc, J., Uehlinger, F.D., 2019. Prevalence, fecal egg counts, and species identification of gastrointestinal nematodes in replacement dairy heifers in Canada. J. DAIRY Sci. 102, 8251–8263. https://doi.org/10.3168/jds.2018-16115
Scott, K., Kelton, D.F., Duffield, T.F., Renaud, D.L., 2019. Risk factors identified on arrival associated with morbidity and mortality at a grain-fed veal facility: A prospective, single-cohort study. J. DAIRY Sci. 102, 9224–9235. https://doi.org/10.3168/jds.2019-16829
Serrano-Martínez, M.E., Cisterna, C.A.B., Romero, R.C.E., Huacho, M.A.Q., Bermabé, A.M., Albornoz, L.A.L., 2019. Evaluation of abortions spontaneously induced by Neospora caninum and risk factors in dairy cattle from Lima, Peru. Rev. Bras. Parasitol. Vet. Braz. J. Vet. Parasitol. Orgao Of. Col. Bras. Parasitol. Vet. 28, 215–220. https://doi.org/10.1590/S1984-29612019026
Småge, S.B., Brevik, Ø.J., Frisch, K., Watanabe, K., Duesund, H., Nylund, A., 2017. Concurrent jellyfish blooms and tenacibaculosis outbreaks in Northern Norwegian Atlantic salmon (Salmo salar) farms. PloS One 12, e0187476. https://doi.org/10.1371/journal.pone.0187476
Söderlund, R., Hakhverdyan, M., Aspan, A., Jansson, E., 2018. Genome analysis provides insights into the epidemiology of infection with flavobacterium psychrophilum among farmed salmonid fish in Sweden. Microb. Genomics 4. https://doi.org/10.1099/mgen.0.000241
Soler-Jiménez, L.C., Paredes-Trujillo, A.I., Vidal-Martínez, V.M., 2017. Helminth parasites of finfish commercial aquaculture in Latin America. J. Helminthol. 91, 110–136. https://doi.org/10.1017/S0022149X16000833
Solomon, D., Shpigel, N.Y., Salamon, H., Goshen, T., 2020. Epidemiology and risk factors of pyelonephritis in Israeli dairy cattle. Isr. J. Vet. Med. 75, 6–11.
Sørensen, J., Vendramin, N., Priess, C., Kannimuthu, D., Henriksen, N.H., Iburg, T.M., Olesen, N.J., Cuenca, A., 2020. Emergence and Spread of Piscine orthoreovirus Genotype 3. Pathog. Basel Switz. 9. https://doi.org/10.3390/pathogens9100823
Svendsen, J.C., Nylund, S., Kristoffersen, A.B., Takle, H., Fossberg Buhaug, J., Jensen, B.B., 2019. Monitoring infection with Piscine myocarditis virus and development of cardiomyopathy syndrome in farmed Atlantic salmon (Salmo salar L.) in Norway. J. Fish Dis. 42, 511–518. https://doi.org/10.1111/jfd.12974
Terceti, M.S., Vences, A., Matanza, X.M., Dalsgaard, I., Pedersen, K., Osorio, C.R., 2018. Molecular Epidemiology of Photobacterium damselae subsp damselae Outbreaks in Marine Rainbow Trout Farms Reveals Extensive Horizontal Gene Transfer and High Genetic Diversity. Front. Microbiol. 9. https://doi.org/10.3389/fmicb.2018.02155
Ulrich, K., Wehner, S., Bekaert, M., Di Paola, N., Dilcher, M., Muir, K.F., Taggart, J.B., Matejusova, I., Weidmann, M., 2018. Molecular epidemiological study on Infectious Pancreatic Necrosis

Virus isolates from aquafarms in Scotland over three decades. J. Gen. Virol. 99, 1567–1581. https://doi.org/10.1099/jgv.0.001155

Urie, N.J., Lombard, J.E., Shivley, C.B., Kopral, C.A., Adams, A.E., Earleywine, T.J., Olson, J.D., Garry, F.B., 2018. Preweaned heifer management on US dairy operations: Part V. Factors associated with morbidity and mortality in preweaned dairy heifer calves. J. DAIRY Sci. 101, 9229–9244. https://doi.org/10.3168/jds.2017-14019

Valldecabres, A., Pires, J.A.A., Silva-Del-Río, N., 2019. Cow-level factors associated with subclinical hypocalcemia at calving in multiparous Jersey cows. J. DAIRY Sci. 102, 8367–8375. https://doi.org/10.3168/jds.2018-16180

Venjakob, P.L., Staufenbiel, R., Heuwieser, W., Borchardt, S., 2019. Serum calcium dynamics within the first 3 days in milk and the associated risk of acute puerperal metritis. J. DAIRY Sci. 102, 11428–11438. https://doi.org/10.3168/jds.2019-16721

Vennerström, P., Välimäki, E., Lyytikäinen, T., Hautaniemi, M., Vidgren, G., Koski, P., Virtala, A.-M., 2017. Viral haemorrhagic septicaemia virus (VHSV Id) infections are detected more consistently using syndromic vs. active surveillance. Dis. Aquat. Organ. 126, 111–123. https://doi.org/10.3354/dao03161

Wang, Yu, Robertson, I.D., Cheng, S., Wang, Yan, Hou, L., Wang, G., Wu, X., Li, X., Chen, Y., Guo, A., 2020. Evaluation of a milk ELISA as an alternative to a serum ELISA in the determination of the prevalence and incidence of brucellosis in dairy herds in Hubei Province, China. Prev. Vet. Med. 182. https://doi.org/10.1016/j.prevetmed.2020.105086

Yang, D.A., Heuer, C., Laven, R., Vink, W.D., Chesterton, R.N., 2017. Farm and cow-level prevalence of bovine digital dermatitis on dairy farms in Taranaki, New Zealand. N. Z. Vet. J. 65, 252–256. https://doi.org/10.1080/00480169.2017.1344587

Yang, D.A., Johnson, W.O., Müller, K.R., Gates, M.C., Laven, R.A., 2019. Estimating the herd and cow level prevalence of bovine digital dermatitis on New Zealand dairy farms: A Bayesian superpopulation approach. Prev. Vet. Med. 165, 76–84. https://doi.org/10.1016/j.prevetmed.2019.02.014

Yugo, D.M., Cossaboom, C.M., Heffron, C.L., Huang, Y.-W., Kenney, S.P., Woolums, A.R., Hurley, D.J., Opriessnig, T., Li, L., Delwart, E., Kanevsky, I., Meng, X.-J., 2019. Evidence for an unknown agent antigenically related to the hepatitis E virus in dairy cows in the United States. J. Med. Virol. 91, 677–686. https://doi.org/10.1002/jmv.25339

Zalcman, E., Burroughs, A., Meyer, A., Mackenzie, C., Sadler, R., Stevenson, M., Ward, M., Gallardo Lagno, A., Happold, J., Cameron, A., Cowled, B., 2021. Sea lice infestation of salmonids in Chile between 2011 and 2017: Use of regulatory data to describe characteristics and identify risk factors. Aquaculture 530, 735752. https://doi.org/10.1016/j.aquaculture.2020.735752
